# PMN-MDSCs-derived exosomal S100A9 drives breast cancer progression by enhancing cancer stemness and CXCL5-mediated metastatic potential

**DOI:** 10.1038/s41420-026-03134-7

**Published:** 2026-05-18

**Authors:** Bo Wang, Binjie Su, Qi Cai, Tao Jiang, Weidong Liu, Xiaoguo Zhao, Yuekang Xu, Changying Guo, Jinyao Li

**Affiliations:** 1 Xinjiang Key Laboratory of Biological Resources and Genetic Engineering, College of Life Science and Technology, Xinjiang University, Urumqi, China; 2 Laboratory Animal Center, Science and Technology Innovation and Transformation Service Center, Xinjiang Medical University, Urumqi, China; 3 Department of Gastroenterology, People’s Hospital of Xinjiang Uygur Autonomous Region, Urumqi, China

**Keywords:** Cancer stem cells, Breast cancer

## Abstract

Polymorphonuclear myeloid-derived suppressor cells (PMN-MDSCs) represent a critical subset of immunosuppressive cells within the tumor microenvironment. Their interaction with breast cancer stem cells (BCSCs) constitutes a vital link that drives the malignant progression of breast cancer, particularly in triple-negative breast cancer (TNBC). However, the precise molecular mechanisms through which these cells mediate BCSC-related stemness reprogramming and enhanced metastatic potential in breast cancer remain inadequately understood. In this study, PMN-MDSCs were isolated from mouse breast cancer solid tumor tissues. Functional characterization was conducted using flow cytometric phenotyping, nuclear morphology analysis, and T cell immunosuppressive function assays. In vivo tumorigenesis experiments confirmed their pro-tumor growth effects and enhanced tumor stem cell characteristics. Concurrently, the CD44^hi^CD24^lo^ subpopulation of 4T1 cells was sorted to systematically verify their tumor stem cell properties and stemness plasticity. In vitro co-culture experiments demonstrated that PMN-MDSCs significantly increased the proportion of CD44^hi^CD24^lo^ stem-like subpopulations in 4T1 cells, prompting non-stem cells to differentiate into stem cell-like cells. They also promoted STAT3 phosphorylation, upregulated the expression of stemness-related proteins and mesenchymal markers, downregulated the epithelial marker E-cadherin, and markedly enhanced CXCL5 secretion. These effects were effectively reversed by the stemness inhibitor Napabucasin. Transwell non-contact co-culture and exosome function assays further confirmed that PMN-MDSCs regulate cancer cell stemness in a paracrine manner. S100A9, a key effector molecule in their exosomes, induced CXCL5 secretion by activating the STAT3 pathway. CXCL5 subsequently activated both the ERK1/2 and STAT3 pathways through autocrine and paracrine mechanisms, enhancing tumor cell metastatic potential and establishing a positive feedback loop that sustains its own secretion. This loop effect was blocked by a CXCL5-neutralizing antibody. Single-cell sequencing data analysis and clinical sample validation of TNBC indicated significantly elevated infiltration of myeloid cells, as well as increased S100A9 and CXCL5 expression levels in cancer tissues, alongside a marked upregulation of the mRNA stemness index. The stemness index was closely associated with poor patient prognosis. In summary, this study reveals that PMN-MDSCs can activate the STAT3-CXCL5-ERK positive feedback regulatory axis via exosomal S100A9, synergistically enhancing breast cancer cell stemness and metastatic capacity. These findings provide a theoretical reference and potential intervention targets for targeting the tumor microenvironment to inhibit TNBC progression.

## Introduction

Cancer stem cells (CSCs) represent a distinct subpopulation within tumors, characterized by their abilities for self-renewal and sustained proliferation [1], which are essential for tumor initiation, progression, metastasis, and recurrence following treatment [2]. The fate of CSCs is influenced by secreted cellular factors and cell-cell interactions within the tumor microenvironment (TME) [3, 4]. In this context, myeloid-derived suppressor cells (MDSCs) create immune-privileged zones by inhibiting the activity of CD8^+^ T cells and natural killer (NK) cells, thereby protecting tumor cells from immune system eradication [5]. Furthermore, MDSCs play a role in maintaining and expanding the CSC pool [6]. For example, elevated levels of nucleus accumbens-associated protein 1 (NAC1) in MDSCs enhance the stemness of TNBC [7]. Conversely, CSCs can promote the proliferation of MDSCs through the secretion of granulocyte-macrophage colony-stimulating factor (GM-CSF) and modulation of aldehyde dehydrogenase 1 (ALDH1) activity, ultimately disrupting T cell immunity and facilitating breast cancer progression [8]. This intricate interplay between MDSCs and CSCs complicates the cancer immune cycle and the formulation of effective tumor treatment strategies.

In the context of cancer, pathologically activated MDSCs are classified into polymorphonuclear cells from the granulocytic lineage (PMN-MDSCs) and monocytic cells (M-MDSCs) [9]. Notably, PMN-MDSCs constitute 70-80% of the total MDSC population, while M-MDSCs typically comprise less than 20% [10]. Upon migration to the TME, M-MDSCs can differentiate into tumor-associated macrophages [11]. Consequently, recent research has increasingly focused on PMN-MDSCs, with heightened levels of PMN-MDSC infiltration significantly correlating with clinical stage, recurrence risk, and poor prognosis in breast cancer [12]. In murine models, PMN-MDSCs are identified by the markers CD11b^+^Ly6G^+/hi^Ly6C^-/int^; in humans, they are characterized by CD11b^+^CD14^-^CD15^+^ or CD11b^+^CD14^-^CD66b^+^ markers [13]. Furthermore, the chemokine receptor CXCR2 is crucial for the recruitment of PMN-MDSCs to the TME, with its primary ligands being CXCL1, CXCL2, and CXCL5 [14].

In tumor immunosuppressive environments, MDSCs primarily target T cells and NK cells. MDSCs inhibit T cell function and promote tumor progression by modulating or depleting critical metabolites such as tryptophan, arginine, and cysteine within the TME [15, 16]. Additionally, MDSCs release exosomes containing GPR84, which induce T cell senescence through the p53 pathway [17]. Targeting VISTA has been shown to alleviate MDSC-mediated T cell suppression [18]. Furthermore, MDSCs express programmed death-ligand 1 (PD-L1) and secrete various soluble factors, including inducible nitric oxide synthase (iNOS), reactive oxygen species (ROS), indoleamine 2,3-dioxygenase (IDO), and arginase-1 (Arg-1), all of which contribute to immune suppression and inhibit NK cell activation [15, 19]. However, tumor development is characterized by a disruption in the balance between immune surveillance and the stemness of mutated cells, with the effect of MDSCs on CSCs remaining inadequately characterized. In this study, we investigated the regulatory influence of PMN-MDSCs on the stemness of the murine breast cancer cell line, 4T1. Our findings demonstrated that co-culturing PMN-MDSCs with breast cancer cell lines enhanced the stemness of 4T1 cells and promoted the expression of CXCL5. We further examined the implications of increased CXCL5 expression on tumor metastasis. Additionally, we discovered that exosomes derived from PMN-MDSCs, along with S100A9 and CXCL5, further enhance the stemness of 4T1 cells. These findings provide novel insights into the mechanisms of interaction between MDSCs and CSCs.

## Results

### The acquisition of PMN-MDSCs and the identification of their activity, morphology, and functions

**Fig. 1:**
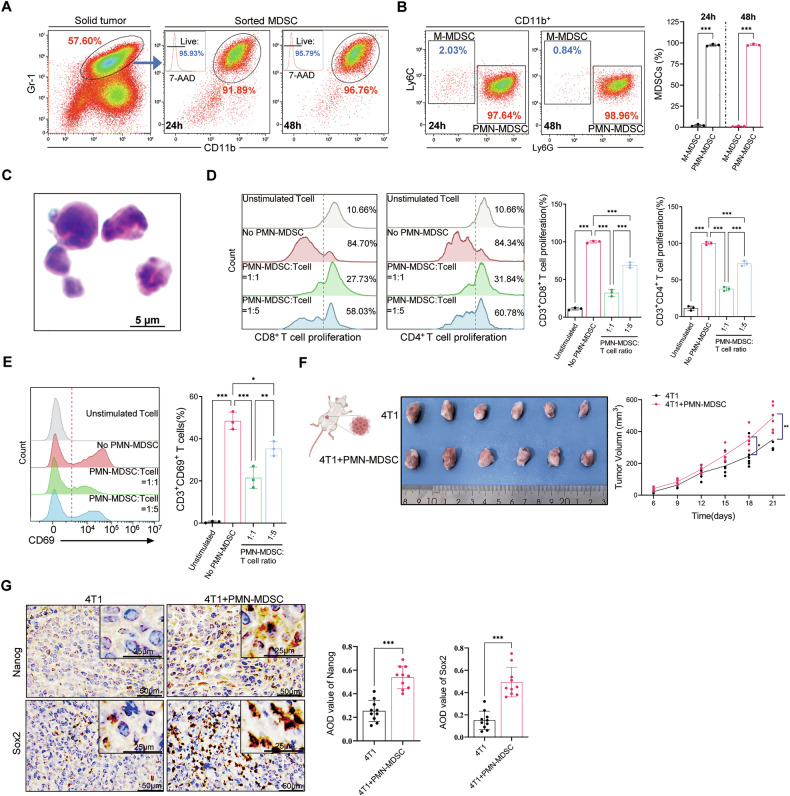
The acquisition of PMN-MDSCs and the identification of their activity, morphology, and function. **A** Representative flow cytometry diagram depicts sorted MDSCs derived from a single-cell suspension prepared from tumors, along with an evaluation of cell viability using 7-AAD staining at both 24 and 48 h. **B** Representative flow cytometry plots and statistical graphs delineate the populations of PMN-MDSCs and M-MDSCs from sorted MDSCs (*n* = 3) at both time points. **C** Wright’s staining demonstrates the morphology of sorted PMN-MDSCs (Representative image). Scale bar: 5 µm. **D** Histogram overlays and quantitative bar charts depict the proliferation of T cells stimulated with CD3/CD28 (without PMN-MDSC), in the absence or presence of PMN-MDSCs at ratios of 1:1 and 1:5 (PMN-MDSC: T cell) for CD8^+^ (left panel; *n* = 3) and CD4^+^ (right panel; *n* = 3) T cells. **E** Histogram overlays and statistical graphs reveal the activation status of T cells treated as described in **D** (*n* = 3). **F** Following the co-injection of PMN-MDSC with 4T1 cells into the fourth mammary fat pad of mice (*n* = 6), tumor volume was measured every three days. On day 21, tumors were excised from euthanized mice, and their sizes were measured (left panel) and plotted (right panel). **G** The results of immunohistochemical staining for stem-related proteins (Nanog and Sox2; *n* = 10) in tumor tissues, accompanied by statistical charts. Scale bar: 50 µm; scale bar: 25 µm. The data are expressed as mean ± SD. The Student’s *t* test is employed for the analysis of two sample groups, while one-way ANOVA followed by Tukey’s post-hoc test was used for the analysis of multiple sample groups. *p < 0.05, **p < 0.01, ***p < 0.001.

As MDSCs increase with tumor progression [20, 21], we aimed to isolate these cells from advanced breast tumor-bearing mice. Initially, we identified the subpopulations of PMN-MDSCs and M-MDSCs in the spleens of tumor-bearing mice using flow cytometry. When the gating strategy for flow cytometry was set to myeloid cells, the results showed that CD11b^+^ PMN-MDSCs constituted the predominant cell population (73.06%), while M-MDSCs represented a smaller proportion (13.11%) (Fig. S1A). Consequently, we referenced the polymorphonuclear position in splenic cells to sort PMN-MDSCs from solid tumors. By adjusting the sorting flow cytometry threshold, we successfully excluded cell debris, small impurities, and mononuclear cells along the forward scatter (FSC) axis, specifically gating PMN-MDSCs (Fig. S1B). The viability of sorted MDSCs at 24 and 48 h was assessed using 7-AAD staining to differentiate between live and dead cells, with results indicating that the survival rate exceeded 95% (Fig. 1A). Subsequently, we identified the populations of PMN-MDSCs and M-MDSCs within the sorted MDSCs. The findings demonstrated that at 24 and 48 h post- sorting, PMN-MDSCs accounted for over 95% of the MDSC population, significantly higher than M-MDSCs (Fig. 1B). The morphology of the sorted PMN-MDSCs was evaluated using Wright’s staining, confirming that all were polymorphonuclear cells (Fig. 1C). These results indicate that our approach, which leverages the localization of PMN-MDSCs in the spleen of tumor-bearing mice and an optimized sorting protocol, is effective.

To validate the functionality of PMN-MDSCs obtained through flow sorting, we co-cultured PMN-MDSCs with CD3/CD28-stimulated T cells at ratios of 1:1 and 1:5. The results indicate that the addition of PMN-MDSCs significantly inhibited the proliferation of both CD8^+^ and CD4^+^ T cells compared to CD3/CD28-stimulated T cells alone. Furthermore, the inhibitory effect of PMN-MDSCs on T cells was more pronounced at a 1:1 ratio than at a 1:5 ratio (Fig. 1D). Validation was further conducted using OT-1 mice, yielding consistent results (Fig. S2A). Notably, when sorted PMN-MDSCs were co-cultured with T cells at both ratios, they effectively inhibited T cell activation, as evidenced by the downregulation of CD69 (Fig. 1E). This indicates that the PMN-MDSCs obtained through sorting possess the ability to suppress T cell proliferation and activation.

With validated immunosuppressive MDSCs in hand, we proceeded to examine their impact on breast cancer growth in vivo. We co-injected PMN-MDSCs alongside the mouse breast cancer cell line 4T1 into the fourth mammary fat pad of mice, while a control group received 4T1 injections alone. Differences between the two groups began to emerge by day 18, with the co-injection group exhibiting larger tumor volumes (Fig. 1F). This difference became more pronounced by day 21, indicating that PMN-MDSCs accelerate tumor progression, consistent with previous reports on MDSCs [6]. Subsequently, we analyzed the expression levels of cancer stemness-related markers Nanog and Sox2 in the tumors of both groups through immunohistochemical assays. The results revealed that the mixed injection group exhibited markedly higher expression of both markers (Fig. 1G), indicating that PMN-MDSCs promote the enhancement of tumor stemness.

### A CD44^hi^CD24^lo^ subpopulation in 4T1 cells exhibits more pronounced stem-like characteristics and possesses differentiation potential

**Fig. 2:**
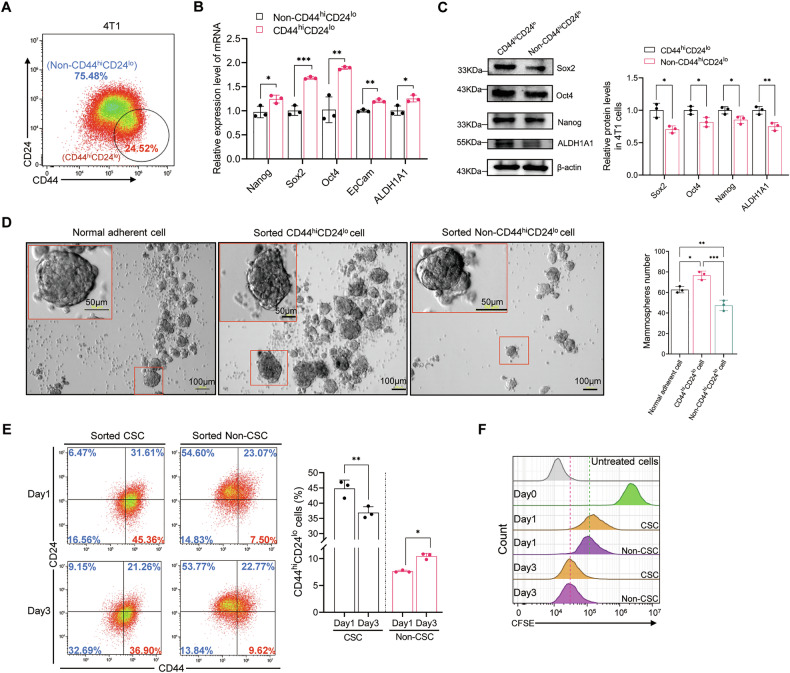
Characterization of CSCs and non-CSCs in 4T1 cells. **A** Representative gating strategy to sort CD44^hi^CD24^lo^ cell population and the non-CD44^hi^CD24^lo^ cell population in 4T1 cells. **B** The relative mRNA expression levels of Nanog, ALDH1A1, Oct4, Epcam, and Sox2 were assessed in both CD44^hi^CD24^lo^ and non-CD44^hi^CD24^lo^ cells (*n* = 3). **C** The results of Western blot experiments, accompanied by quantitative analysis, indicating that Sox2, Oct4, Nanog, and ALDH1A1 are expressed (*n* = 3). **D** Representative images and quantitative analysis of Mammosphere formation were conducted in low-attachment 6-well plates under serum-free conditions (*n* = 3). **E** Representative flow cytometry plots illustrating the CSCs and non-CSCs sorted from 4T1 cells on days 1 and 3 of in vitro culture, along with the statistical results of the CD44^hi^CD24^lo^ cell population (*n* = 3). **F** Representative histogram overlay demonstrated the proliferation rates of CFSE-labeled CSCs and non-CSCs on days 1 and 3. Here, CSCs refer to the sorted CD44^hi^CD24^lo^ cells, while non-CSCs refer to the non- CD44^hi^CD24^lo^ cells. The data are expressed as mean ± SD. The Student’s t-test is employed for the analysis of two sample groups, while one-way ANOVA followed by Tukey’s post-hoc test was used for the analysis of multiple sample groups. *p < 0.05, **p < 0.01, ***p < 0.001.

The stem-promoting effect of 4T1 cells by PMN-MDSCs in vivo prompted us to investigate the underlying mechanisms in vitro. Initially, we identified cancer cell populations exhibiting characteristics of CSCs, specifically the CD44^hi^CD24^lo^ cell populations, which demonstrated traits typical of tumor stem cells [22, 23]. We sorted CD44^hi^CD24^lo^ and non-CD44^hi^CD24^lo^ cells from the 4T1 cells using fluorescence-activated cell sorting (FACS) and subsequently compared their stemness (Fig. 2A). The results of the relative mRNA expression levels indicated that, compared to the non-CD44^hi^CD24^lo^ cells, the CD44^hi^CD24^lo^ cells exhibited significantly higher expression of stem cell markers such as Nanog, Sox2, Oct4, EpCam, and ALDH1A1 (Fig. 2B). Protein examination data from Western blot analyses for Sox2, Oct4, Nanog, and ALDHA1 in the CD44^hi^CD24^lo^ cell population corroborated the RNA findings (Fig. 2C). Additionally, the mammosphere formation assay demonstrated that the CD44^hi^CD24^lo^ cell population exhibited a higher sphere-forming rate, indicating enhanced self-renewal capacity (Fig. 2D). Collectively, these results support the notion that the sorted CD44^hi^CD24^lo^ cell population exhibits characteristics akin to CSCs. In this context, we refer to the sorted CD44^hi^CD24^lo^ cells as CSCs and the non-CD44^hi^CD24^lo^ cells as non-CSCs.

To investigate the stability of CD44^hi^CD24^lo^ stem cells in the 4T1 culture, sorted CSCs and non-CSCs were continuously cultured in vitro. The results indicated that, compared to day one post-sorting, the proportion of the CD44^hi^CD24^lo^ cell population in the CSC group significantly decreased by day three (Fig. 2E). In contrast, the proportion of the CD44^hi^CD24^lo^ cell population in the non-CSC group significantly increased (Fig. 2E). These findings suggest that CD44^hi^CD24^lo^ cells within CSCs undergo differentiation over time, whereas cells in the non-CSC group also differentiate into CD44^hi^CD24^lo^ cells. To determine whether these percentage changes were due to proliferation or differentiation, we labeled the sorted cells with CFSE and monitored their proliferation rates on days one and three. The results demonstrated that the proliferation rates of CSCs and non-CSCs were comparable, showing no significant difference on either day (Fig. 2F). Collectively, these findings indicate the plasticity of stemness in breast cancer cells; over time, CD44^hi^CD24^lo^ cells can differentiate into non-CSCs, while non-CSCs can also differentiate into CD44^hi^CD24^lo^ cells.

### PMN-MDSCs enhance the stemness characteristics of 4T1 cells

Given the plasticity of sorted CD44^hi^CD24^lo^ cells, we aimed to investigate the impact of PMN-MDSCs on their differentiation by co-culturing sorted PMN-MDSCs with 4T1 cells at various ratios.

**Fig. 3:**
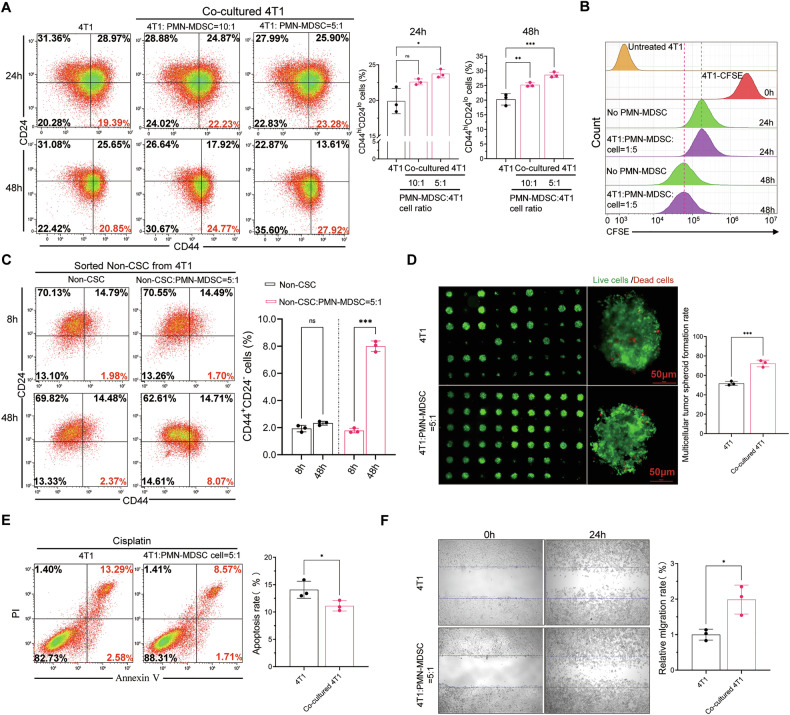
The role of PMN-MDSCs in enhancing the stemness characteristics of 4T1 cells. **A** Representative flow cytometry plots and corresponding statistical graphs illustrate the proportion of CD44^hi^CD24^lo^ cells in 4T1 cells, as well as in 4T1 cells co-cultured with PMN-MDSCs at ratios of 10:1 and 5:1, measured at 24 and 48 h (*n* = 3). **B** Representative histogram overlay of the proliferation rate of CFSE-labeled 4T1 cells after co-culture with PMN-MDSCs is presented. **C** Representative flow cytometry graphs and bar charts illustrate the changes in the CD44^hi^CD24^lo^ cell population within the non-CSCs (non-CD44^hi^CD24^lo^ cells) subpopulation isolated from 4T1 cells after co-culturing with PMN-MDSCs at a ratio of 5:1 for 8 h and 48 h, along with the statistical results (*n* = 3). **D** Representative fluorescence images and statistical graphs illustrate the spheroid formation rates of 4T1 cells co-cultured with PMN-MDSCs in a chip matrix, with Calcein-AM staining live cells in green and PI staining dead cells in red. Scale bar: 50 µm; *n* = 3. **E** Flow cytometry plots and statistical graphs demonstrate the apoptosis rates of 4T1 cells co-cultured with PMN-MDSCs and treated with cisplatin (5 µM for 24 h; *n* = 3). **F** Representative images and statistical graphs depict the migration rates of 4T1 cells co-cultured with PMN-MDSCs following a cell scratch assay after 24 h (*n* = 3). The data are expressed as mean ± SD. The Student’s t-test is employed for the analysis of two sample groups, while one-way ANOVA followed by Tukey’s post-hoc test was utilized for the analysis of multiple sample groups. *p < 0.05, **p < 0.01, ***p < 0.001, with “ns” indicating no significance.

The results indicate that a 5:1 ratio of 4T1 to PMN-MDSCs leads to a significant increase in the CD44^hi^CD24^lo^ cell population compared to the control group after 24 and 48 h (Fig. 3A). However, the 10:1 ratio did not show significant changes at 24 h (Fig. 3A). We labeled 4T1 cells with CFSE and observed the proliferation rates of 4T1 cells co-cultured with PMN-MDSCs compared to control 4T1 cells over a period of 48 h. The results indicated that the proliferation rates of the two groups of 4T1 cells were comparable, thus eliminating the possibility of differences in cell population ratios due to variations in cell proliferation rates (Fig. 3B). This suggests that the presence of PMN-MDSCs leads to an increase in the population of CD44^hi^CD24^lo^ cells within the 4T1 cells. Furthermore, we co-cultured other mouse breast cancer cell lines with PMN-MDSCs at a 5:1 ratio for 48 h and obtained consistent results, including EMT6, EO771, and PY8119 (Fig. S3A). Therefore, to investigate whether this impact on the stemness of breast cancer cells was unique to PMN-MDSCs, we isolated polymorphonuclear cells from the mammary fat pad tissue of healthy mice and added them separately to 4T1 cells along with PMN-MDSCs isolated from tumors. The results indicated that only the CD44^hi^CD24^lo^ cell population in 4T1 cells co-cultured with PMN-MDSCs exhibited a significant increase (Fig. S3B). Additionally, non-CSCs isolated from 4T1 cells were co-cultured with PMN-MDSCs, revealing that PMN-MDSCs significantly enhanced the increase of the CD44^hi^CD24^lo^ cell population in non-CSC cells (Fig. 3C). Collectively, these findings indicated that PMN-MDSCs can promote the stemness of breast cancer cells by facilitating the differentiation of non-CD44^hi^CD24^lo^ cell populations into CD44^hi^CD24^lo^ cell populations in 4T1 cells.

Additionally, we employed a chip-based cell culture technique to investigate the impact of PMN-MDSCs on tumor cell spheroid formation using the Calcein-AM/PI dual staining reagent (Fig. S3C). The results demonstrated that co-culturing PMN-MDSCs with 4T1 cells significantly enhanced the rate of multicellular tumor spheroid formation (Fig. 3D). This finding suggests that PMN-MDSCs facilitate the development of multicellular tumor spheroids. Furthermore, we assessed the sensitivity of 4T1 cells in the PMN-MDSCs co-culture group to the cytotoxic effects of the anti-cancer drug cisplatin. The results indicated that the apoptosis rate of 4T1 cells in the co-culture group was significantly lower than that in the control group (Fig. 3E). This finding suggests that PMN-MDSCs promote the stemness of 4T1 cells and enhance their resistance to chemotherapy drugs. Notably, we observed that 4T1 cells co-cultured with PMN-MDSCs exhibited improved migratory ability, indicating that the presence of PMN-MDSCs may influence tumor cell metastasis (Fig. 3F).

### PMN-MDSCs enhance the stemness of 4T1 cells and promote their secretion of CXCL5 by activating the STAT3 signaling pathway

**Fig. 4:**
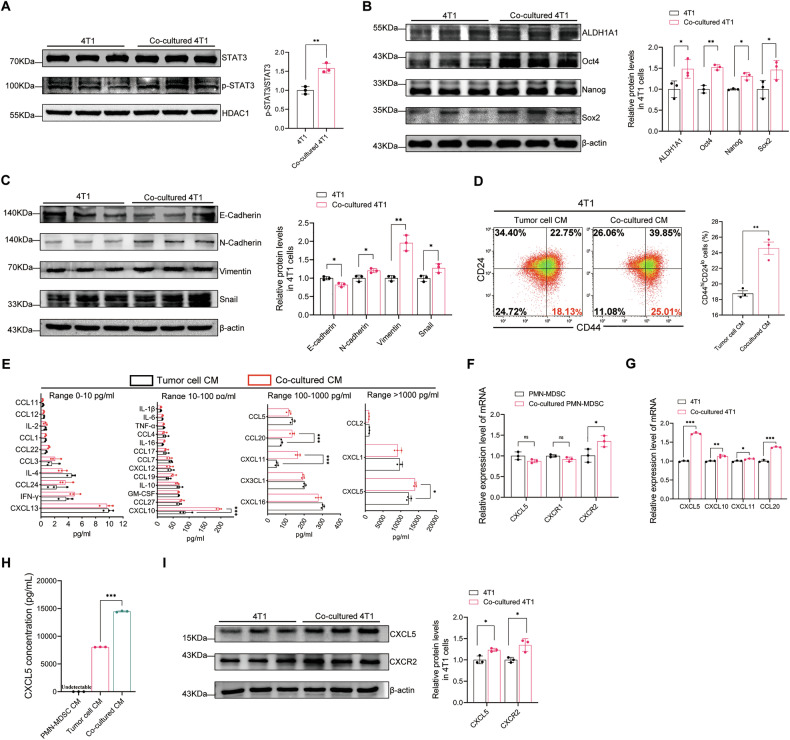
PMN-MDSCs enhance the stemness of 4T1 cells by activating the STAT3 signaling pathway, which promotes the secretion of CXCL5 from 4T1 cells. **A** Western blot analysis and its quantitative evaluation reveal the expression levels of STAT3 and p-STAT3 in 4T1 cells cultured with or without PMN-MDSCs (*n* = 3). **B** Western blotting and its quantitative analysis demonstrate the expression of ALDH1A1, Oct4, Nanog, and Sox2 in both 4T1 cells and their co-cultured counterparts with PMN-MDSCs (*n* = 3). **C** Western blotting and its quantitative analysis reveal the expression levels of E-Cadherin, N-Cadherin, Vimentin, and Snail in 4T1 cells under co-culture conditions with and without PMN-MDSCs (*n* = 3). **D** Representative flow cytometry plots and statistical graphs illustrating the proportion of CD44^hi^CD24^lo^ cells cultured for 48 h in 4T1 cell conditioned medium (CM) and in the co-culture CM of 4T1 with PMN-MDSC (*n* = 3). **E** The expression levels of the Mouse Chemokine Panel 31-Plex in tumor cell CM and co-cultured CM were evaluated, with results categorized into four groups: 0-10 pg/mL, 10-100 pg/mL, 100-1000 pg/mL, and greater than 1000 pg/mL (*n* = 3). **F** The relative mRNA expression levels of CXCL5, CXCR1, and CXCR2 were evaluated between PMN-MDSCs co-cultured with 4T1 cells and PMN-MDSCs cultured alone (*n* = 3). **G** The relative mRNA expression levels of CXCL5, CXCL10, CXCL11, and CCL20 between 4T1 cells and co-cultured 4T1 cells with PMN-MDSCs were evaluated (*n* = 3). **H** The expression of CXCL5 in the conditioned media collected from PMN-MDSCs, 4T1 cells, and the co-culture of 4T1 cells with PMN-MDSCs was detected using ELISA (*n* = 3). **I** Western blot analysis and its quantitative evaluation show the expression levels of CXCL5 and CXCR2 in 4T1 cells cultured with and without PMN-MDSCs (*n* = 3). The data are expressed as mean ± SD. The Student’s *t* test is employed for the analysis of two sample groups, while one-way ANOVA followed by Tukey’s post-hoc test was utilized for the analysis of multiple sample groups. *p < 0.05, **p < 0.01, ***p < 0.001, with “ns” indicating no significance.

To investigate the impact of PMN-MDSCs on the expression of the STAT3 protein in 4T1 cells, which is linked to tumor cell stemness, we conducted a Western blot analysis. The results revealed that the relative expression level of p-STAT3 in co-cultured 4T1 cells was significantly higher than that in the control group of 4T1 cells alone (Fig. 4A). This finding indicates that PMN-MDSCs further activate the STAT3 signaling pathway in 4T1 cells. Additionally, we analyzed other proteins associated with tumor cell stemness through Western blot analysis. The results demonstrated that ALDH1A1, Oct4, Nanog, and Sox2 were all significantly upregulated in the co-culture group compared to the control group (Fig. 4B). This suggests that PMN-MDSCs enhance the expression of stemness-related proteins in 4T1 cells. Given the association of the STAT3 signaling pathway with tumor cell stemness, we hypothesize that PMN-MDSCs enhance STAT3 activation, thereby promoting the increased expression of stemness-related proteins in 4T1 cells.

Furthermore, we examined the key proteins associated with epithelial-mesenchymal transition in this group. Western blot analysis revealed that the levels of the epithelial cell marker E-Cadherin in the co-culture group were significantly lower than those in the control group, while the levels of the mesenchymal markers N-Cadherin, Vimentin, and Snail were significantly higher (Fig. 4C). This finding indicates a reduction in the epithelial phenotype of 4T1 cells in the co-culture group, suggesting an increased metastatic potential. Additionally, we collected cells from the conditioned medium (CM) after 48 h (Fig. S4A). After the removal of PMN-MDSCs (CD11b^+^ cells), no significant differences were observed in the expression of CD44^hi^CD24^lo^ between the two groups of suspended 4T1 cells (CD11b^-^ cells) (Fig. S4B). This observation rules out the possibility that cancer cells exhibiting enhanced stemness are non-adherent cells.

Subsequently, we collected the supernatant from the co-culture of 4T1 cells and PMN-MDSCs, which we designated as co-cultured conditioned medium (Co-cultured CM). The supernatant from the 4T1 cells served as a control. The Co-cultured CM was then reintroduced into the dish, and the 4T1 cells were cultured under identical conditions. The results indicated that the CM from the co-culture significantly increased the proportion of CD44^hi^CD24^lo^ cell populations in 4T1 cells compared to the control group (Fig. 4D). This finding suggests that the CM from the co-culture can regulate the stemness of 4T1 cells, potentially due to the biological mediators released by PMN-MDSCs. We compared the expression levels of cytokines and chemokines between the co-culture group and the tumor cell CM using the Mouse Chemokine Panel 31-Plex assay. The results indicated significant differences in the levels of CXCL5, CXCL10, CXCL11 and CCL20 in the co-culture group of 4T1 cells compared to the control group (Fig. 4E), with CXCL5 exhibiting notably high expression. This finding suggests that the increased protein levels in the CM of the co-culture may result from either the secretion of PMN-MDSCs or enhanced secretion by the 4T1 cells themselves.

To differentiate this, we first assessed PMN-MDSCs and found no significant differences in the expression levels of CXCL5 and the CXCR1 receptor when cultured alone or co-cultured with 4T1, except for CXCR2 (Fig. 4F). These results suggest that the elevated expression of CXCL5 in the CM may be attributed to the co-cultured 4T1 cells, indicating that 4T1 cells enhance the secretion of CXCL5 following co-culture. Next, we evaluated the relative mRNA expression levels of 4T1 cells between the two groups. The results showed that the relative mRNA levels of the CXCL5, CXCL10, CXCL11, and CCL20 genes in the co-cultured 4T1 group were significantly higher than those in the control group (Fig. 4G). Given the consistency of results from the Mouse Chemokine Panel and the mRNA levels for CXCL5, along with its increased secretion, we further confirmed that the level of CXCL5 in the co-culture group was higher than that in the control group, but it was undetectable in the PMN-MDSCs cultured alone (Fig. 4H). Subsequently, we examined the protein expression levels of CXCL5 and its receptor, CXCR2. The results indicated that both CXCL5 and CXCR2 were significantly upregulated in 4T1 cells from the co-culture group compared to the control group (Fig. 4I). This observation suggests that PMN-MDSCs promote the enhancement of CXCL5 secretion in 4T1 cells, which acts on themselves through the CXCR2 receptor. Furthermore, the tumor stemness-related protein Sox2 is known to bind to the promoter region of CXCL5, thereby positively regulating its expression [24]. These findings indicate that PMN-MDSCs enhance the stemness of 4T1 cells, leading to increased CXCL5 secretion.

### CXCL5 interacts with the CXCR2 receptors expressed by 4T1 cells, establishing an autocrine loop that activates the ERK signaling pathway and enhances metastatic capability

**Fig. 5:**
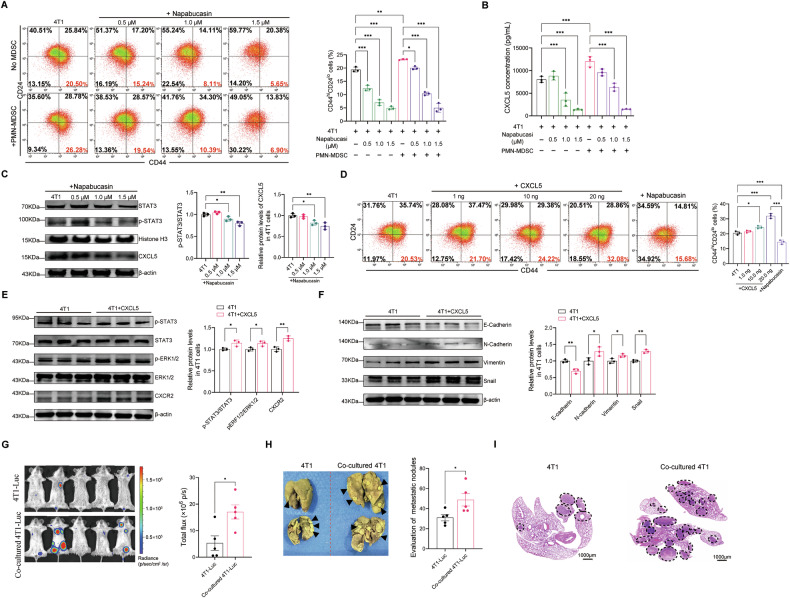
The addition of exogenous CXCL5 activates the ERK signaling pathway in 4T1 cells, thereby enhancing the metastatic potential of tumor cells. **A** Representative flow cytometry plots and bar graphs depict the proportion of CD44^hi^CD24^lo^ cells in 4T1 cells and those co-cultured with PMN-MDSCs, treated with Napabucasin at concentrations of 0.5 μM, 1.0 μM, and 1.5 μM (*n* = 3). **B** The ELISA was performed to assess the effect of Napabucasin at the aforementioned concentrations on CXCL5 expression in the conditioned medium (CM) of 4T1 cells and 4T1 cells co-cultured with PMN-MDSCs (*n* = 3). **C** Western blotting and its quantitative evaluation analysis revealed the levels of STAT3, p-STAT3, and CXCL5 in 4T1 cells and 4T1 cells treated with Napabucasin at concentrations of 0.5 μM, 1.0 μM, and 1.5 μM (*n* = 3). **D** Representative flow cytometry plots and bar graphs demonstrate the impact of exogenous CXCL5 protein at concentrations of 1 ng/ml, 10 ng/ml, and 20 ng/ml on the proportion of CD44^hi^CD24^lo^ cells in 4T1 cells (*n* = 3). **E** Western blotting and its quantitative evaluation analysis showed the expression levels of STAT3, p-STAT3, ERK1/2, p-ERK1/2, and CXCR2 in 4T1 cells and those treated with 20 ng/ml of CXCL5 (*n* = 3). **F** Western blotting and its quantitative evaluation results indicated the expression levels of E-Cadherin, N-Cadherin, Vimentin, and Snail in 4T1 cells and those treated with 20 ng/ml of CXCL5 (*n* = 3). **G** Bioluminescent imaging was utilized to evaluate the metastatic burden in mice intravenously injected with 1 × 10^5^ 4T1-Luc cells or co-cultured 4T1-Luc cells (*n* = 5). **H** The lung tissues of mice bearing breast cancer were evaluated after treatment with Bouin’s fixative, and a statistical assessment of inflammatory metastatic nodules was conducted (Representative images; *n* = 5). **I** HE staining was performed on lung tissue sections obtained from breast cancer-bearing mice (Representative images; *n* = 5). Scale bars: 1000 μm. The data are expressed as mean ± SD. The Student’s t-test is employed for the analysis of two sample groups, while one-way ANOVA followed by Tukey’s post-hoc test was utilized for the analysis of multiple sample groups. *p < 0.05, **p < 0.01, ***p < 0.001.

We selected Napabucasin, a well-known inhibitor of tumor cell stemness, and incorporated it into the co-culture system at varying concentrations based on the IC_50_ values (Fig. S5A). The results indicate that the addition of 0.5 μM, 1.0 μM, and 1.5 μM of Napabucasin significantly reduced the population of CD44^hi^CD24^lo^ cells within 4T1 cells (Fig. 5A). Furthermore, a significant decrease in the population of CD44^hi^CD24^lo^ cells in 4T1 cells was also observed when co-cultured with PMN-MDSCs (Fig. 5A). This suggests that as the concentration of Napabucasin increases, its inhibitory effect on the stemness of tumor cells becomes more pronounced. Subsequently, we assessed the alterations in CXCL5 levels in the conditioned medium (CM) following the addition of Napabucasin. The findings indicated that the incorporation of 0.5 μM, 1.0 μM, and 1.5 μM Napabucasin significantly inhibited the secretion of CXCL5 by 4T1 cells (Fig. 5A). Similarly, in the co-culture system with PMN-MDSCs, Napabucasin markedly decreased the secretion of CXCL5 by 4T1 cells (Fig. 5B). This indicates that the inhibition of stemness in 4T1 cells positively correlates with a reduction in CXCL5 secretion. At the protein level, upon the addition of 1.0 μM and 1.5 μM Napabucasin, the relative expression level of p-STAT3 was inhibited, and the protein expression of CXCL5 was also suppressed (Fig. 5C). Notably, Napabucasin can also inhibit the release of ROS from PMN-MDSCs (Fig. S5B), which aligns with the finding that Napabucasin eliminates the immunosuppressive capabilities of MDSCs [25].

To establish the bilateral relationship between 4T1 stemness and CXCL5, we supplemented the 4T1 cell culture medium with exogenous CXCL5 and monitored the changes in the CD44^hi^CD24^lo^ cell population within the 4T1 cells. The results indicated that the addition of 20 ng/ml of CXCL5 protein resulted in a significant increase in the CD44^hi^CD24^lo^ cell population compared to the control group lacking CXCL5 (Fig. 5D), suggesting that CXCL5 protein enhances the stemness of 4T1 cells.

In the CXCL5-supplemented 4T1 group, the levels of CXCR2, along with the relative expressions of p-STAT3 and p-ERK1/2, were significantly elevated compared to those in the control group (Fig. 5E). Subsequently, we administered the CXCR2 inhibitor SB225002 to the 4T1 cell culture at concentrations of 0.5 μM, 1.0 μM, and 1.5 μM. The results indicated that both the 1.0 μM and 1.5 μM concentrations of SB225002 significantly reduced the CD44^hi^CD24^lo^ cell population in 4T1 cells, suggesting that the inhibition of CXCR2 expression suppresses the stemness of tumor cells (Fig. S6A). Furthermore, in the presence of exogenous CXCL5 protein, SB225002 also inhibited the stemness of 4T1 cells (Fig. S6B). When a neutralizing antibody against CXCL5 was introduced, the presence of exogenous CXCL5 protein did not influence the stemness of 4T1 cells (Fig. S6C). In 4T1 cells supplemented with exogenous CXCL5, we observed a significant decrease in E-Cadherin levels, while the protein expressions of N-Cadherin, Vimentin, and Snail were significantly increased (Fig. 5F). However, when CXCL5-neutralizing antibodies were added to eliminate CXCL5, there was no significant difference in the relevant proteins compared to 4T1 cells cultured alone (Fig. S6D). In contrast, when SB225002 was added to block the CXCR2 receptor, a significant increase in E-Cadherin levels was observed, while the protein expressions of N-Cadherin, Vimentin, and Snail were significantly decreased (Fig. S6D). These findings suggest that the CXCL5-CXCR2 axis is a key driver of stemness in 4T1 cells, indicating that CXCL5 binds to the CXCR2 receptor, enhancing the stemness of 4T1 cells and activating the ERK pathway to promote metastasis.

To investigate tumor cell metastasis, we injected 4T1-Luc cells and co-cultured them with PMN-MDSCs into the tail veins of BALB/c mice. Prior to injection, we confirmed that the 4T1-Luc cells co-cultured with PMN-MDSCs exhibited a significantly higher population of CD44^hi^CD24^lo^ cells (Fig. S7A). Additionally, we ensured that the injected 4T1-Luc cells were free from PMN-MDSC interference (Fig. S7B). The results indicated that the 4T1-Luc cells co-cultured with PMN-MDSCs demonstrated enhanced metastatic capability (Fig. 5G). Furthermore, we observed that the co-culture group displayed significantly more inflammatory foci compared to the control group (Figs. 5H and S7C). Similar results were obtained from the HE staining of lung tissue sections (Fig. 5I). These findings suggest that PMN-MDSCs promote the enhancement of stemness in breast cancer cells, thereby increasing their metastatic potential.

### Exosomes derived from PMN-MDSCs enhance the stemness of 4T1 cells, promoting their secretion of CXCL5

**Fig. 6:**
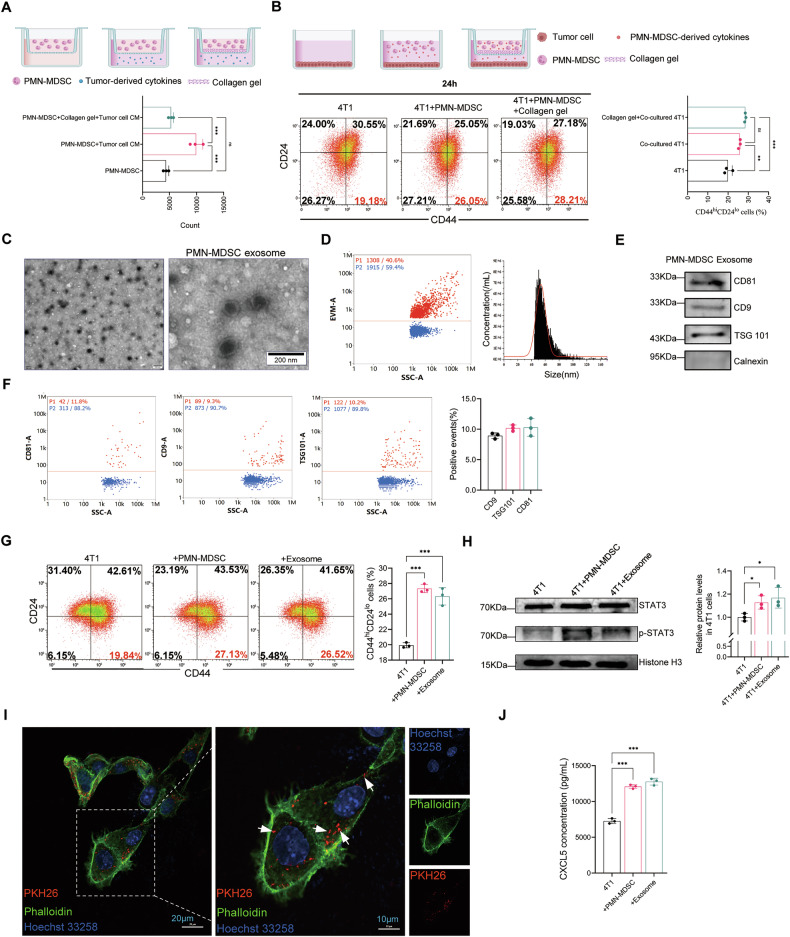
Identification of exosomes derived from PMN-MDSCs and their capacity to penetrate 4T1 cells, enhancing the stemness of these cells. **A** The schematic diagram in the top panel outlines three groups within the lower chamber of the Transwell: one with normal medium, another with conditioned medium (CM) from tumor cells (4T1), and a third group with tumor cell CM supplemented with collagen gel coated in the insert. The cell count graph in the bottom panel displays the number of PMN-MDSCs that migrated to the lower chamber 24 h post-introduction into the Transwell insert (*n* = 3). **B** The schematic diagram in the top panel illustrates three groups under Transwell chambers containing normal medium with 4T1 cells: one without PMN-MDSC, one with PMN-MDSC, and one with PMN-MDSC in chambers coated with collagen gel. After 24 h, the 4T1 cells in the bottom were recovered and analyzed to assess the proportion of CD44^hi^CD24^lo^ cells among the three groups, as shown in the representative flow cytometry scatter plot in the left bottom panel and the bar graph in the right bottom panel (*n* = 3). **C** The representative morphological structure of PMN-MDSC-derived exosomes was examined using transmission electron microscopy (Representative image). **D** Results from nano-flow cytometry demonstrated the particle size distribution of PMN-MDSC-derived exosome membrane structures, which were labeled with Extracellular Vesicle Membrane Red Stains. **E** Western blot analyses confirmed the presence of CD81, CD9, and TSG101 in PMN-MDSC-derived exosomes (*n* = 3). **F** The results of nano-flow cytometry and the statistical analysis of the positive ratio of PMN-MDSC-derived exosomes labeled with CD81, CD9, and TSG101 are presented (*n* = 3). **G** Representative flow cytometry results, along with a proportional bar graph illustrating the presence of CD44^hi^CD24^lo^ cells in 4T1 cells co-cultured with PMN-MDSC and PMN-MDSC-derived exosomes, are shown (*n* = 3). **H** Western blotting and its quantitative results for STAT3 and p-STAT3 in 4T1 cells co-cultured with PMN-MDSC and PMN-MDSC-derived exosomes are provided (*n* = 3). **I** Representative fluorescence images of PHK26-labeled PMN-MDSC-derived exosomes co-cultured with 4T1 cells are presented, with PHK26 depicted in red, phalloidin in green, and nuclei in blue (*n* = 3). Scale bars measure 10 µm and 20 µm. **J** ELISA results for the detection of CXCL5 secretion by 4T1 cells co-cultured with PMN-MDSC and PMN-MDSC-derived exosomes are reported (*n* = 3). The data are expressed as mean ± SD. One-way ANOVA followed by Tukey’s post-hoc test was utilized for the analysis of multiple sample groups. The Student’s *t* test is employed for the analysis of two sample groups. *p < 0.05, **p < 0.01, ***p < 0.001, with ‘ns’ indicating no significance.

Given that PMN-MDSCs are non-adherent cells while 4T1 cells grow in an adherent state, we hypothesize that PMN-MDSCs may influence 4T1 cells through the release of specific soluble mediators. Notably, the tumor cell conditioned medium (CM), derived from the supernatant of 4T1 cell culture, exhibited chemotactic effects on PMN-MDSCs [26]. We utilized a collagen gel coated in a Transwell chamber to partially block PMN-MDSCs. The media released by PMN-MDSCs can permeate through the pores of the collagen gel and enter the lower chamber. The results indicated that in the two groups with tumor cell CM in the lower chamber, the group with the collagen gel blocking effect exhibited significantly lower diffusion of PMN-MDSCs compared to the normal Transwell. However, the degree of PMN-MDSCs diffusion in the collagen gel group was comparable to that in the lower chamber without tumor cell CM (normal culture medium), indicating no significant difference (Fig. 6A). This suggests that the collagen gel inhibited the chemotactic effect of the CM on PMN-MDSCs, resulting in diffusion patterns akin to normal conditions. Consequently, we investigated whether PMN-MDSCs would influence changes in the CD44^hi^CD24^lo^ cell population of adherent 4T1 cells following the addition of the collagen gel barrier. The results revealed that, compared to the control group’s 4T1 CD44^hi^CD24^lo^ cell population, the population in the collagen gel group significantly increased, exhibiting a statistically significant difference (Fig. 6B). This finding suggests that PMN-MDSCs can modulate the stemness of adherent 4T1 cells by releasing biological mediators, thereby affecting the stemness population of 4T1 cells in a non-contact manner.

We collected exosomes from PMN-MDSCs to investigate their potential role as mediators. Initially, we confirmed that the exosomes derived from PMN-MDSCs are membrane-enclosed structures (Fig. 6C). Subsequently, we analyzed the contents and particle sizes of these exosomes using the Flow NanoAnalyzer. The results indicated that 40.6% of the measured particles exhibited membrane structures, with particle sizes predominantly ranging from 45 to 70 nm (Fig. 6D). We verified the expression of exosomal marker proteins CD81, CD9, and TSG101 in these particles through Western blot analysis (Fig. 6E). Furthermore, the proportion of CD81, CD9, and TSG101 was demonstrated via nanoflow cytometry (Fig. 6F). Notably, during the detection of the marker proteins, the particle size of the exosomes remained consistent within the range of 45 to 70 nm (Fig. S8). Collectively, these findings confirm that we have successfully collected exosomes secreted by PMN-MDSCs.

Subsequently, we investigated the co-culture of exosomes derived from PMN-MDSCs with 4T1 cells. The results demonstrated a significant increase in the CD44^hi^CD24^lo^ cell population among 4T1 cells in the exosome-added co-culture group (Fig. 6G). Additionally, Western blot analysis revealed an enhanced activation of p-STAT3 (Fig. 6H). These findings suggest that PMN-MDSCs may influence 4T1 cells via exosomes, thereby augmenting the stemness of 4T1 cells. To further substantiate our hypothesis, we employed confocal laser scanning microscopy to observe the internalization of exosomes into the cells, confirming that PKH26-labeled exosomes successfully entered the cells to mediate the effects of PMN-MDSCs (Fig. 6I). Furthermore, we evaluated the expression levels of CXCL5 in the supernatant, which indicated that the levels in both the PMN-MDSC and exosome groups were significantly higher than those in the 4T1 monoculture group (Fig. 6J). These findings imply that exosomes originating from PMN-MDSCs enhance the stemness of 4T1 cells, subsequently promoting the secretion of CXCL5 by 4T1 cells.

### S100A9 in PMN-MDSC exosomes promotes the stemness of 4T1 cells and enhances their secretion of CXCL5

**Fig. 7:**
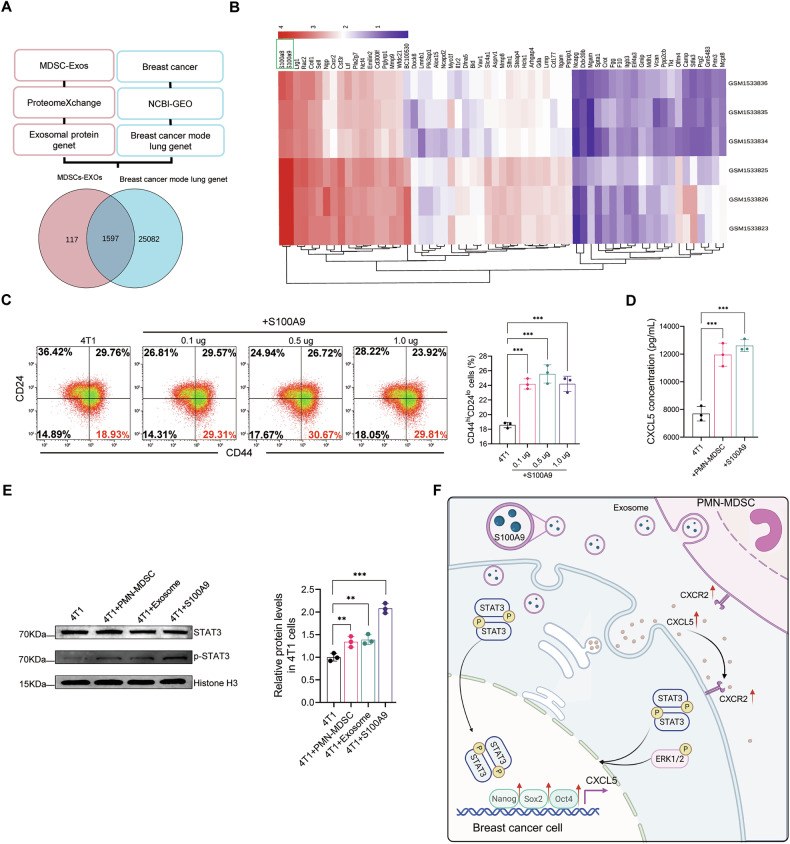
S100A9 is a crucial protein found in exosomes derived from PMN-MDSCs, which enhances the stemness of 4T1 cells and increases their secretion of CXCL5. **A** The Venn diagram is presented, derived from the analysis of protein gene data from MDSC exosomes and pre-metastatic lung gene data. **B** The cluster heatmap analysis of co-expressed genes is shown. **C** Representative flow cytometry plots and bar graphs demonstrate the effects of exogenous S100A9 protein at concentrations of 0.1 µg/mL, 0.5 µg/mL, and 1.0 µg/mL on the proportion of CD44^hi^CD24^lo^ cells in 4T1 cells (*n* = 3). **D** ELISA results reveal the expression levels of CXCL5 in 4T1 cells treated with S100A9 or conditioned medium from PMN-MDSCs (*n* = 3). **E** Western blotting and its quantitative evaluation analysis reveals the levels of STAT3 and p-STAT3 in 4T1 cells co-cultured with PMN-MDSCs, PMN-MDSC-derived exosomes, and S100A9 (*n* = 3). **F** The schematic model illustrates that exosomes secreted by PMN-MDSCs (containing S100A9) influence the stemness of 4T1 cells via the STAT3 signaling pathway, thereby promoting CXCL5 secretion, which provides positive feedback to further enhance stemness. The data are expressed as mean ± SD. One-way ANOVA followed by Tukey’s post-hoc test was utilized for the analysis of multiple sample groups. The Student’s t-test is employed for the analysis of two sample groups. **p < 0.01, ***p < 0.001.

By matching the protein gene data of MDSC-Exosomes, which contains 1714 genes (PXD006204-2), with the pre-metastatic lung gene data of breast cancer, comprising 26,679 genes (GDS5437), a Venn diagram revealed 1597 overlapping data points (Fig. 7A). Among these, S100A8 and S100A9 were identified as highly expressed genes (Fig. 7B). To verify the biological effects of the transcriptional products of these two genes on breast cancer cells, we added exogenous recombinant proteins from the S100A8 and S100A9 genes to the culture medium of 4T1 cells. The results indicated that concentrations of 0.1 μg/ml, 0.5 μg/ml, and 1.0 μg/ml of S100A9 significantly increased the CD44^hi^CD24^lo^ cell population in 4T1 cells (Fig. 7C). In contrast, the same concentrations of S100A8 did not affect the CD44^hi^CD24^lo^ cell population (Fig. S9A), suggesting that S100A9 specifically enhances the stemness of 4T1 cells. Additionally, we incorporated S100A9 into other mouse breast cancer cell lines, including EMT6, EO771, and PY8119, and obtained similar results (Fig. S9B). We then examined the expression of CXCL5 in the supernatant of the co-culture group of 4T1 cells with S100A9, revealing a significant increase in CXCL5 expression (Fig. 7D). Similarly, CXCL5 levels in the supernatants of EMT6, EO771, and PY8119 cells yielded consistent results, although the expression level of CXCL5 in EO771 cells was relatively low (Fig. S9C). Western blot analysis demonstrated that S100A9 promotes the activation of p-STAT3/STAT3 (Fig. 7E). Collectively, these data suggest that S100A9 in the exosomes of PMN-MDSCs is a crucial factor in enhancing the stemness of 4T1 cells.

### An increase in myeloid cells, elevated expression of S100A9, and enhanced stemness are correlated with poor prognosis in TNBC

**Fig. 8:**
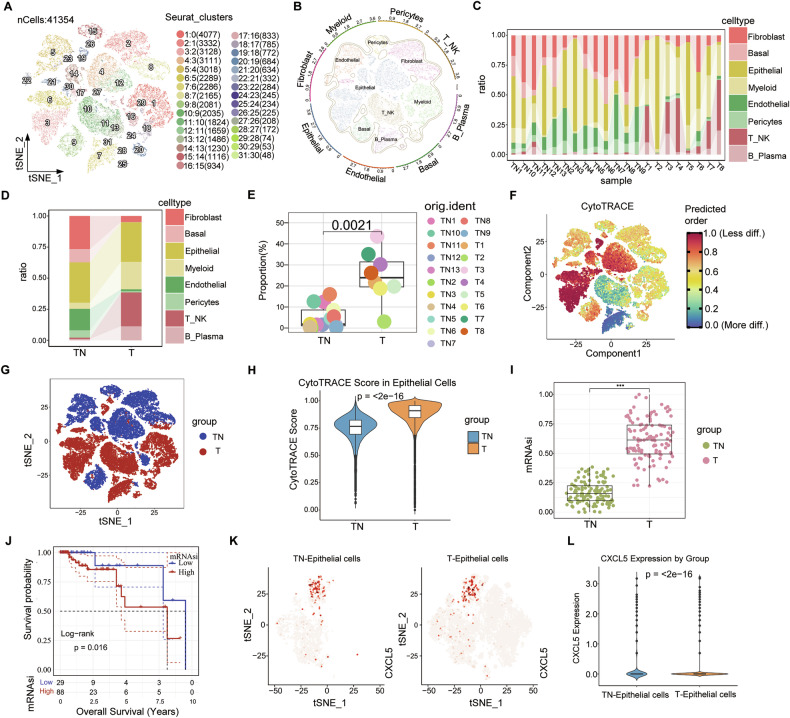
The analysis of the single-cell transcriptome atlas and stemness in TNBC. **A** The t-SNE dimensionality reduction clustering distribution map illustrates that 41,354 cells have been categorized into 31 Seurat clusters. **B** The annotations correspond to 8 distinct cell subsets within the breast cancer tumor microenvironment. **C** The bar chart depicts the proportions of various cell types in 13 TN samples and 8 T samples. **D** Another bar chart compares the proportions of cell types between TN and T. **E** The proportion of myeloid cells in TN and T is highlighted. **F** The tSNE dimensionality reduction clustering distribution map displays cell stemness as calculated by cytoTRACE. **G** The tSNE dimensionality reduction clustering distribution map of TN and T is provided. **H** The violin plot illustrates the cytoTRACE scores of epithelial cells in both TN and T. **I** A box plot presents the mRNAsi for TNBC. **J** Kaplan–Meier survival curves are shown for overall survival in TNBC patients categorized by high and low mRNAsi. **K** The tSNE dimensionality reduction clustering distribution map of CXCL5 expression in TN- epithelial cells and T- epithelial cells is included. **L** The violin plot displays the expression levels of CXCL5 in TN-Epithelial cells and T-Epithelial cells. TN: tumor-adjacent normal tissue; T: tumor tissue. The data are expressed as mean ± SD. The Student’s t-test is employed for the analysis of two sample groups. ***p < 0.001.

We subsequently analyzed scRNA-seq data from TNBC, categorizing 41,354 cells within the tumor microenvironment into 31 Seurat clusters (Fig. 8A). These clusters were further annotated into eight distinct cell subsets, including dominant epithelial cells (comprising basal types), myeloid immune cells, stromal components such as fibroblasts, endothelial cells, and pericytes, as well as lymphocytes, which include T/NK and B/plasma cells (Fig. 8B). Following this, we conducted a comprehensive analysis of the proportions of these cell subsets in samples from tumor-adjacent normal tissue (TN) and tumor tissue (T) (Fig. 8C, D), revealing a significant increase in the proportion of myeloid cells in T (Fig. 8E). The levels of tumor-infiltrating myeloid cells are significantly elevated in breast cancer patients, and the increase in myeloid cells also leads to a rise in PMN-MDSCs [27]. We employed the cytoTRACE algorithm to assess the degree of cellular stemness (Fig. 8F) and performed t-distributed stochastic neighbor embedding (tSNE) dimensionality reduction clustering for TN and T (Fig. 8G). The results indicated that in epithelial cells, the cytoTRACE score of T was significantly higher than that of TN (Fig. 8H), suggesting that malignant tumor epithelial cells exhibit greater stemness. Subsequently, we calculated the stemness score of bulk RNA-seq data using the OCLR algorithm based on specific stem cell probes. The results demonstrated that the mRNA expression‑based stemness index (mRNAsi) of T was significantly higher than that of TN (Fig. 8I). Moreover, the group with high mRNAsi exhibited significantly worse overall survival (Fig. 8J). We then selected epithelial cells for tSNE dimensionality reduction clustering to analyze the expression of CXCL5 (Fig. 8K). The results revealed that CXCL5 expression in T was significantly elevated (Fig. 8L). These findings indicate that cells in breast tumors possess greater stemness and significantly poorer overall survival, with malignant epithelial cells in tumors secreting more CXCL5.

**Fig. 9:**
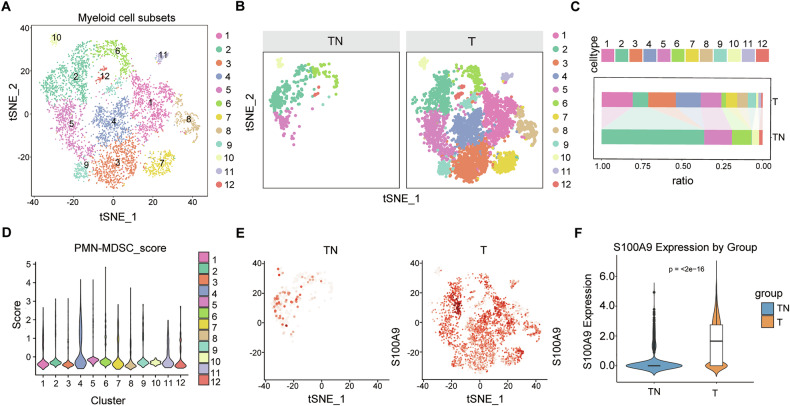
A comprehensive analysis of myeloid cells within the single-cell transcriptome atlas of TNBC. **A** The t-SNE dimensionality reduction technique reveals distinct subpopulations of myeloid cells. **B** A t-SNE clustering of myeloid cells under TN and T conditions is illustrated. **C** A bar chart depicts the proportions of various cell types within the myeloid cell population. **D** A violin plot illustrates the scores of PMN-MDSCs across different myeloid cell subpopulations. **E** The t-SNE clustering again highlights the distribution of S100A9 in TN and T. **F** A violin plot demonstrates the expression levels of S100A9 in both TN and T conditions. TN: tumor-adjacent normal tissue; T: tumor tissue. The data are expressed as mean ± SD. The Student’s *t* test is employed for the analysis of two sample groups. ***p < 0.001.

Further analysis was conducted on myeloid cells. The t-SNE dimensionality reduction technique revealed 12 subsets of myeloid cells (Fig. 9A). The subsets of myeloid cells with higher abundance in T are illustrated in Fig. 9B. Compared to TN, additional myeloid cell subsets 1, 3, 4, 7, 8, 9, and 11 were increased in T (Fig. 9C). Scoring using a gene set composed of PMN-MDSC markers, such as CD84, PTGER2, CD52, CXCR1, and CXCR2, indicated that the 4-cluster had the highest score (Fig. 9D). We subsequently performed tSNE dimensionality reduction visualization of S100A9 in TN and T (Fig. 9E), revealing that the expression of S100A9 in T was significantly higher than that in TN (Fig. 9F). These results indicate that, compared to TN, myeloid cells in breast tumors are significantly increased and secrete more S100A9.

### mIHC revealed a high expression of PMN-MDSCs, S100A9, and CXCL5 in breast cancer tumor tissues

**Fig. 10:**
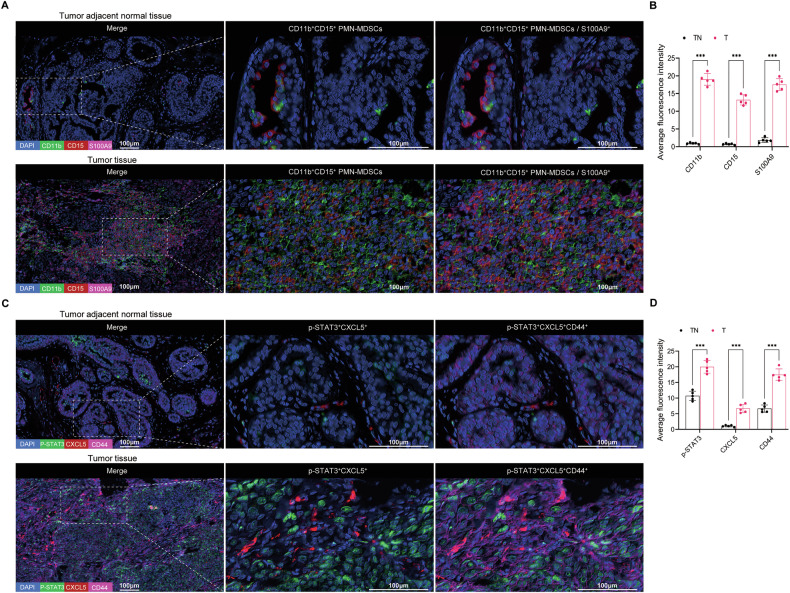
Representative immunofluorescence images illustrating the infiltration of PMN-MDSCs, S100A9, and CXCL5 in tumor tissues of triple-negative breast cancer, as well as in adjacent normal tissues. **A** The representative fluorescence images depict PMN-MDSCs (CD11b^+^CD15^+^) and S100A9 within breast cancer tissues compared to adjacent normal tissues. DAPI is shown in blue, CD11b in green, CD15 in red, and S100A9 in pink. **B** A statistical graph displays the average fluorescence intensity of CD11b, CD15, and S100A9 in both TN and T (*n* = 5). **C** Representative fluorescence images of p-STAT3, CXCL5, and CD44 in breast cancer tissues and adjacent normal breast tissues are presented. DAPI is shown in blue, p-STAT3 in green, CXCL5 in red, and CD44 in pink. **D** A statistical graph illustrates the average fluorescence intensity of p-STAT3, CXCL5, and CD44 in TN and T (*n* = 5). Scale bars: 100 μm. TN: tumor-adjacent normal tissue; T: tumor tissue. The data are expressed as mean ± SD. The Student’s t-test is employed for the analysis of two sample groups. ***p < 0.001.

We conducted a comprehensive analysis of the expression levels of PMN-MDSCs (CD11b^+^CD15^+^) and S100A9 in tumor tissue samples obtained from patients diagnosed with TNBC, utilizing Multiplex immunofluorescence (mIHC) technology (Fig. 10A). The statistical analysis revealed a significant increase in the expression levels of CD11b, CD15, and S100A9 within the tumor tissue (Fig. 10B). This finding indicates a marked increase in the infiltration of PMN-MDSCs into the tumor microenvironment, along with a notable elevation in S100A9 levels. Furthermore, we assessed the expression of p-STAT3, CXCL5, and CD44 in the tumor tissue (Fig. 10C). The statistical results demonstrated a significant upregulation of p-STAT3, CXCL5, and CD44 in the tumor samples (Fig. 10D). These observations suggest an enhancement in tumor cell stemness, an elevated activation level of STAT3, and a potential for metastasis, aligning with previous experimental findings.

## Discussion

The TME comprises various non-malignant cell types embedded within the extracellular matrix [28]. In this context, MDSCs are recruited by CSCs, facilitating the evasion of immune surveillance. MDSCs can sustain the stemness of tumor cells through several mechanisms. For instance, elevated levels of NAC1 in MDSCs support the stemness of TNBC [7]. Furthermore, CCL20-modulated PMN-MDSCs enhance the self-renewal capabilities of breast cancer cells through the CXCL2-CXCR2 pathway. Conversely, CSCs also exert a significant influence on MDSCs; for example, the developmental signals of MDSCs are regulated by CSCs [29]. Additionally, CSCs promote the expansion of MDSCs by secreting GM-CSF, which reduces T cell immunity and promotes the progression of breast cancer [8]. Collectively, a complex crosstalk exists between PMN-MDSCs and CSCs within the TME.

In the current study, we initially considered two common methods for obtaining PMN-MDSCs: positive selection from the spleens of tumor-bearing mice using Ly6G magnetic beads [30] and inducing the differentiation of precursor cells from mouse bone marrow into PMN-MDSCs [30, 31]. However, recognizing that PMN-MDSCs in the TME are recruited, pathologically activated, and exist in their most authentic state, we refined our gating strategy for flow cytometry, successfully isolating PMN-MDSCs from single-cell suspensions prepared from solid tumors with a purity exceeding 95% (Fig. 1B). This inhibitory effect aligns with that of PMN-MDSCs isolated from the primary tumors of MMTV-PyMT spontaneous breast cancer mice[32]. Subsequently, the sorted PMN-MDSCs were co-injected with 4T1 cells into the mammary fat pads of mice. By day 18, differences were observed between the two groups, with the mixed injection group exhibiting larger tumor volumes and accelerated tumor progression over time (Fig. 1F). These results are consistent with those observed in MCF-7 and MDSC co-injections[6]. Additionally, we observed that PMN-MDSCs promote the enhancement of tumor cell stemness in vivo (Fig. 1G).

Subsequent in vitro experiments demonstrated that co-cultivation of 4T1 cells with PMN-MDSCs significantly increased the proportion of the CD44ⁱʰCD24ˡᵒ stem cell subpopulation (Fig. 3A). This phenomenon was reproducible across multiple mouse breast cancer cell lines, including EMT6, EO771, and PY8119 (Fig. S3A), suggesting that PMN-MDSCs enhance the stemness of breast cancer cells. Mechanistically, following co-cultivation, the phosphorylation level of STAT3 in 4T1 cells was significantly upregulated, accompanied by an increase in the expression of stemness-related proteins and mesenchymal markers, as well as a notable enhancement in CXCL5 secretion (Fig. 4A–I). The intensity of CXCL5 expression correlates with the degree of malignancy, metastatic potential, and inflammatory infiltration [33]. Conversely, a reduction in CXCL5 levels may decrease the number of immunosuppressive myeloid cells, thereby promoting the infiltration and activation of CD8^+^ T cells, which enhances anti-tumor immunity and influences the metastatic potential of tumors [34]. The expression of CXCL5 is positively regulated by the tumor stemness protein Sox2 [24]. Treatment of 4T1 cells with the stemness inhibitor Napabucasin significantly reduced the proportion of stem-like cell subpopulations and suppressed CXCL5 secretion by 4T1 cells (Fig. 5C), indicating that PMN-MDSCs enhance the stemness of 4T1 cells and promote CXCL5 secretion by activating the STAT3 pathway. Further studies revealed that both the co-culture supernatant and exogenous CXCL5 protein could enhance the stemness of cancer cells (Fig. 5D). CXCL5 was shown to upregulate the levels of CXCR2, p-STAT3, and p-ERK1/2 in 4T1 cells, promoting the expression of mesenchymal markers (Fig. 5E, F). This suggests that CXCL5 enhances the metastatic potential of tumor cells by activating the ERK pathway, and the use of a CXCL5 neutralizing antibody could reverse these effects. In conclusion, CXCL5 secreted by 4T1 cells, promoted by PMN-MDSCs, maintains breast cancer cell stemness and metastasis through the STAT3/ERK signaling axis, forming a positive feedback loop that amplifies CXCL5 secretion, thereby continuously strengthening the malignant phenotype of the tumor.

The CXCL5-CXCR2 axis plays a pivotal role in stimulating tumor growth, angiogenesis, and the proliferation and dissemination of breast cancer cells within the bone [35, 36]. The expression and activation of CXCR2 in breast cancer cells correlate with enhanced stemness [37]. Treatment of tumor-bearing mice with the CXCR2 antagonist SX-682 resulted in inhibited tumor growth and a reduction in MDSCs within the tumor microenvironment, while the CXCR2 antagonist SB265610 effectively inhibited lung metastasis in these mice [38, 39]. Furthermore, blocking CXCR2 with the antagonist SB225002 not only inhibits cancer cell proliferation but also prevents CXCL5-induced signaling [36]. In this study, SB225002 was found to inhibit the stemness of breast cancer cells and reduce the metastatic potential of adenocarcinoma cells (Fig. S6B, D). Additionally, the CXCL5-CXCR2 axis can recruit immunosuppressive cells such as PMN-MDSCs for infiltration, suppress anti-tumor immune responses, promote angiogenesis, and provide a favorable microenvironment for tumor progression [40].

The Immunosuppressive mechanisms of PMN-MDSCs towards targeted cells involve both contact-dependent and non-contact modes of action, which vary according to the pathological context and the type of target cells. For example, PMN-MDSCs enhance their inhibitory effects through direct interactions with T cells via surface molecules such as PD-L1 and VISTA, which are recognized as immune checkpoint molecules, or through the binding of adhesion molecules like CD11b/CD18 with target cells, including CAR T cells [41, 42]. Additionally, PMN-MDSCs can secrete inhibitory cytokines (e.g., Arg1, Nos2, ROS) or exosomes that transmit immunosuppressive signals [43]. In the present study, we found that our acquired PMN-MDSCs can influence the stemness of 4T1 cells in a non-contact manner, with similar effects observed from the CM obtained through co-culture of 4T1 cells with PMN-MDSCs (Fig. 6B). Furthermore, the exosomes derived from PMN-MDSCs, particularly the S100A9 present in these exosomes, were found to significantly promote the activation of p-STAT3/STAT3 (Figs. 6H and 7E), suggesting that PMN-MDSCs secrete exosomes that facilitate the expansion of tumor stem cells in a non-contact manner, with the highly expressed S100A9 in exosomes serving as a critical factor. The concentration gradient of recombinant S100A9 used in this study has a clear physiological relevance, aligning with the reported physiological levels of S100A9 in the serum of cancer patients and the enrichment status in patients with recurrence [44]. Consistently, overexpression of S100A9 has been reported to correlate positively with markers of tumor stem cells [45]. Following their uptake by tumor cells, exosomes derived from PMN-MDSCs can increase the expression of stem cell markers in these cells, promote tumor spheroid formation, and enhance the stemness of colorectal cancer cells [46]. Furthermore, as a chemokine, S100A9 can attract more PMN-MDSCs to the tumor site, thereby enhancing the immunosuppressive effect [47]. Within the TME, PMN-MDSCs can secrete S100A9, establishing a positive feedback loop that amplifies their tumor-promoting effects [48], which not only exacerbates immunosuppression but may also indirectly promote the self-renewal of tumor stem cells by influencing signaling pathways and cytokine networks.

Through the mining of the scRNA-seq database of TNBC, a comprehensive analysis was conducted on the proportions of various cell subpopulations in tumor-adjacent normal tissue (TN) and tumor tissue (T) samples (Fig. 8C, D). The results indicated a significant increase in the proportion of myeloid cells in T (Fig. 9E). Furthermore, the mRNAsi in T was markedly higher than that in TN (Fig. 8I), and the expression of CXCL5 in T was significantly elevated (Fig. 8L). Additionally, the overall survival of the high mRNAsi group was notably poorer (Fig. 8J). Further analysis of myeloid cells revealed that the highest score in the 4-cluster analysis corresponded to the PMN-MDSC (Fig. 9D). Myeloid cells in tumor tissue exhibited significantly higher levels of S100A9 (Fig. 9F). Subsequently, a considerable increase in the infiltration of PMN-MDSCs within the tumor tissue was observed in patient TNBC tumor sample slices. The levels of S100A9 were also significantly elevated (Fig. 10B), and the expressions of p-STAT3, CXCL5, and CD44 were notably reviewed (Fig. 10D). These findings are consistent with the experimental results observed in vitro.

This study has several limitations. First, the silencing experiment of S100A9 in primary PMN-MDSCs was not completed due to challenges in cell transfection and maintaining cellular activity. Second, the intravenous injection-based in vivo metastasis model does not fully replicate the process of spontaneous tumor dissemination. Third, the precipitation method used for isolating exosomes poses a risk of minor contamination. Furthermore, the dynamics of receptor interactions following CXCR2 upregulation, as well as the role of tumor-derived CXCL5 in bone marrow recruitment, were not thoroughly explored. In future research, we aim to refine the mechanisms of this study by optimizing methodologies, technologies, and models to address these limitations.

Collectively, this study demonstrates that PMN-MDSCs enhance the stemness of breast cancer cells through the secretion of exosomes, wherein the S100A9 protein activates the STAT3 signaling pathway in breast cancer cells, leading to an increased secretion of CXCL5 and thereby promoting tumor metastasis. These findings contribute to a deeper understanding of the complex crosstalk between PMN-MDSCs and CSCs, providing a theoretical basis for the design of therapeutic strategies in clinical settings.

## Materials and methods

### Cell culture

The mouse breast cancer cell lines 4T1, EMT6, EO771, PY8119, and the human breast cancer cell line MDA-MB-231 were obtained from the original cell seed bank of the research group. Routine monitoring for potential mycoplasma contamination is conducted for all cell lines within the laboratory. The 4T1-Luc cell line was generously provided by Dr. Lei Zhang from the College of Life Science and Technology at Xinjiang University. The cells were cultured at 37 °C in a 5% CO2 incubator using RPMI 1640 medium (Gibco, USA, 11875093) or DMEM medium (Gibco, USA, 11875135), both of which were supplemented with 10% fetal bovine serum (FBS) (TIANHANG, China, 13011-8611), 100 units/mL penicillin, and 100 μg/mL streptomycin (Gibco, USA, 15070063).

### Mice and tumor models

Five- to six-week-old female BALB/c mice were obtained from the Animal Experiment Center of Xinjiang Medical University, while OT-I mice were sourced from Jackson Laboratory. All mice were housed at the Animal Experiment Center of Xinjiang Medical University. Animal experiments were conducted in accordance with the protocols approved by the Animal Ethics Committee of Xinjiang Medical University. Mice were randomly divided into groups of six animals each. A total of 5 × 10^5^ 4T1 cells were inoculated into the fourth mammary gland fat pads. Tumor growth was monitored every three days, and tumor volume was calculated using the formula: V = 0.5 × length × width². On day 21, the mice were euthanized, and their spleens were collected for flow cytometry analysis; tumors were harvested for pathological examination and PMN-MDSCs isolation. Additionally, mice were randomly divided into groups of five animals each, and 1×10^5^ 4T1 or 4T1-Luc cells were injected into the tail vein of BALB/c mice. On day 18, following the administration of D-Luciferin potassium (Mec, USA, 115144-35-9), lung metastasis was observed using a small animal in vivo imaging system (PerkinElmer Health Sciences, USA, IVIS).

### RNA isolation and quantitative real-time PCR

Follow the instructions provided in the RNA extraction kit (Takara, Japan, 9109) to dissolve the final pellet in 50 µl of RNase-free water. Synthesize complementary DNA (cDNA) using the PCR instrument (Applied Biosystems, USA, Veriti™) according to the steps outlined in the RT-PCR kit (Takara, Japan, RR036A). ConFig. The reaction system is based on the guidelines of the TB Green® Premix Ex Taq™ II reagent, and performs quantification using the QuantStudio™ 3 Real-Time PCR System (Applied Biosystems, USA). The primers employed for quantitative reverse transcription PCR (qRT-PCR) are detailed in Supplementary Table S1.

### Flow cytometry assay

For the flow cytometry detection of CXCL5, first, add 2 µl of Protein Transport Inhibitor (Becton Dickinson, USA, 554724) for every 3 mL of cell culture. After 6 h, collect the cells and follow the instructions provided in the intracellular fixation and permeabilization buffer kit (Invitrogen, USA, 88-8824) to sequentially incubate with the CXCL5 antibody and the corresponding secondary antibody, followed by machine detection. Additionally, when analyzing cancer cells, utilize anti-mouse CD44 and CD24 antibodies. For immune cell analysis, antibodies against mouse CD45, CD11b, CD3, CD4, CD8, and CD69 were employed. Cell viability was assessed using 7-AAD or DAPI staining. Furthermore, the antibody information for flow cytometry is presented in Supplementary Table S2.

### Isolation and identification of PMN-MDSCs

Tumor tissues from mice were separated and minced using scissors. The tissues were subsequently digested in PBS containing 0.25 mg/mL collagenase IV (Gibco, USA, 17104019), 20 U/mL DNase I (Sigma, USA, 10104159001), and 2% FBS. The cell mixture was placed on a shaker and incubated at 37 °C for 30 min. Following this incubation, the tumor particles were vigorously pipetted with a 3 mL Pasteur pipette to facilitate dissociation. The resulting digested cell mixture was filtered through a 70 μm cell strainer to obtain a single-cell suspension. This suspension was treated with anti-mouse CD16/CD32 antibody (BioLegend, USA, 101302) at a concentration of 1 µg per 10^6^ cells for 15 min, followed by resuspension and centrifugation. Subsequently, CD45, CD11b, and Ly6G/6 C antibodies were added and incubated for 20 min. DAPI was then introduced, and the threshold was adjusted using a flow sorter (Beckman, USA, CytoFLEX SRT) to sort PMN-MDSCs. Cell morphology was assessed using Wright’s staining, while the activity and differentiation of MDSCs were evaluated using DAPI, Ly6G antibody, and Ly6C antibody via the flow cytometer (Beckman, USA, CytoFLEX).

### T-cell proliferation suppression assays

Spleens from female BALB/c mice and OT-1 mice were utilized to prepare single-cell suspensions. The procedures were conducted following the manufacturer’s instructions for Dynabeads® Untouched™ Mouse T Cells (Invitrogen, USA, 11413D). The isolated untouched mouse T cells were labeled with 1 mmol/L CFSE (Invitrogen, USA, 65-0850-84) and subsequently seeded into 48-well plates. PMN-MDSCs, obtained through flow cytometry sorting, were mixed and co-cultured with T cells in a specific ratio. T cells isolated from BALB/c mice were activated using Dynabeads® Mouse T-Activator CD3/CD28 (Gibco, USA, 11453D), while T cells from OT-1 mice were activated with 0.5 ng/mL SIINFEKL peptide (Sigma, USA, S7951). The inhibitory effects on T cell proliferation were evaluated by measuring CFSE fluorescence intensity using flow cytometry.

### Mammosphere formation assay

Seed 1 × 10^4^ cells per well in a low-attachment 6-well cell culture plate (Corning, USA, 3471). The cells were cultured in DMEM/F12 medium supplemented with 2% B27 (Gibco, USA, 17504044), 10 ng/mL bFGF (PeproTech, USA, 450-33-01 M), 20 ng/mL EGF (PeproTech, USA, 315-09-500UG), 2% KnockOut (Gibco, USA, 10828010), 0.4% BSA (Biofroxx, Germany, 4240GR100), 5 µg/mL insulin-transferrin-selenium (Gibco, USA, 41400045), 2 mM/100 mL GlutaMAX™ (Gibco, USA, 35050061), 100 units/mL penicillin, and 100 µg/mL streptomycin. The complete medium of the Mammosphere should be replaced with a fresh one every three days.

### Co-culture system and conditioned medium

To begin, collect 4T1 cells and plate 1.5 × 10^5^ cells in a 12-well plate or 6 × 10^5^ cells in a 60 mm culture dish. Subsequently, add PMN-MDSCs in a predetermined proportion. Following a 48-h culture period, collect the co-cultured cells, label them with flow cytometry antibodies, and conduct flow cytometry analysis. Additionally, centrifuge the CM and filter it using a 0.22 µm filter device (Millipore, USA, SLGP033RB). Depending on the experimental group, add either complete medium, CM, or seed 4T1 cells into the lower chamber of the Transwell (Corning, USA, 3422). Prepare rattail tendon collagen type I (Solarbio, China, C8062) by mixing it with NaOH, followed by thorough mixing with PBS. Coat the resulting mixture onto the chamber of a Transwell and allow the gel to solidify at room temperature for 20 min. After solidification, add 550 µL of complete medium containing PMN-MDSCs. After 24 h, collect the cells from the lower chamber medium, label them with the Ly6G flow cytometry antibody, and quantify them using a flow cytometer.

### Tumor spheroids in the chip matrix

The chip measures 2 × 3 cm and contains eight small matrices, each comprising 11 rows and 11 columns. The micro-wells of this chip matrix have a diameter of 100 µm, with each small matrix capable of accommodating an array of 121 spheroids. Cells are collected, counted, and introduced to the chip using the hanging drop method. After settling the cells, the medium is added, and the chip is incubated. Following the formation of breast tumor spheroids, a staining solution is prepared according to the protocol provided in the Calcein-AM/PI Cell Viability/Cytotoxicity Assay Kit (Biosharp, China, BL130A). This solution is then added to the array, and staining is observed using an inverted fluorescence microscope (Nikon, China, ECLIPSE Ts2).

### Mouse multiplex panel assay

The procedure was conducted in accordance with the instructions provided by the Bio-Plex Pro Mouse Chemokine Panel 31-Plex kit (BIO-RAD, USA, 12009159). Prior to the experiment, all reagents were equilibrated at room temperature for 30 min. Subsequently, 50 μl of beads, standards, quality controls, and samples were added to the wells and incubated at 800 rpm for 1 h at room temperature. Following this incubation, the beads were washed, and 50 μl of detection antibody was added, followed by another incubation at 800 rpm for 30 min at room temperature. After a subsequent wash, 50 μl of PE-streptavidin was added and incubated at 800 rpm for an additional 30 min at room temperature. After washing the beads again, 100 μl of sheath fluid was added and incubated at room temperature while shaking at 800 rpm for 2 min. Detection was performed using the Liquid Suspension Chip Analysis System (Luminex, USA, X-200).

### Extraction and identification of exosomes

To extract and identify exosomes, collect the supernatant of PMN-MDSCs cultured in exosome-specific medium for 48 h. Following the instructions provided with the exosome extraction kit (Duolaimi, China, DL21082), centrifuge the sample at 10,000 g for 10 min to eliminate cell debris. Next, add the Exosome Concentration Solution and centrifuge again at 10,000 × *g* for 60 min at 4 °C, discarding the supernatant afterward. Resuspend the precipitate and purify it using column filtration. For imaging, pipette 15 μl of the exosome sample onto a copper grid, add 3% phosphotungstic acid negative staining solution (Phygene, China, PH1309), and stain at room temperature for 1 min, followed by several washes. After air-drying the sample, observe and capture images using a transmission electron microscope (JEOL, Japan, JEM-F200).

For the analysis of PMN-MDSC-derived exosomes (PMN-MDSC-Exo), further dilute the sample in 1 ml of PBS. Utilize the NanoFCM (China, U30E) to assess their concentration and particle size distribution. Label the lipid membrane structure with Extracellular Vesicle Membrane Red Stains (NanoFCM, China, NEPUL-638), and combine the exosome samples with flow cytometry antibodies CD81 and CD9, incubating at 37 °C for 20 min. Following this, incubate the TSG101 antibody with the exosome samples, then apply a fluorescent secondary antibody for an additional 20 min before analyzing the samples using Nano-flow Cytometry. The expression levels of exosome markers (CD81, CD9, TSG101, and Calnexin) were determined via Western blot analysis.

### Immunofluorescence

To perform immunofluorescence, PKH26 dye is added to the exosomes and incubated at 37 °C for 5 min. The staining is then terminated by adding serum without exosomes, followed by the re-isolation of the exosomes at 16,000 × *g* for 1 h. The exosomes are subsequently resuspended in 100 μL of PBS. Following this, 4T1 cells are plated into confocal-specific culture dishes (NEST, China, 801022) and cultured overnight until they reach 50–70% confluency. Fluorescence-labeled exosomes are added, and the cells are cultured for an additional 24 h. After discarding the cell culture medium, the cells are fixed and subjected to sequential staining with phalloidin and Hoechst 33258. Images are captured using a laser confocal microscope (Nikon, Japan, A1R HD25).

### Western blot

Cells or exosomes were collected, and total protein was extracted from each sample using RIPA lysis buffer (Beyotime, China, P0013B), supplemented with protease inhibitor (Beyotime, China, P1005) and phosphatase inhibitor (Beyotime, China, P1081). The protein concentration in the samples was determined using the BCA protein assay kit (Thermo Scientific, USA, 23227). The samples were then mixed with loading buffer and heated at 100 °C for 10 min; however, exosomes do not require heating. Proteins were separated by SDS-PAGE and subsequently transferred to a PVDF membrane (Merck, Germany, IPVH00010). After blocking the membrane in blocking buffer for 2 h, it was incubated with the primary antibody (refer to Supplementary Table S2) overnight at 4 °C. Following this, the membrane was incubated with the secondary antibody (refer to Supplementary Table S2) at 37 °C for 1 h. Protein detection was performed using the LAS 4000 system (Fujifilm, Japan). Protein bands were analyzed using a hypersensitive chemiluminescence (ECL) detection reagent (Biosharp, China, BL520A). The original data from the Western blot are provided in the supplementary materials.

### Acquisition and pre-processing of scRNA-seq data

The Gene Expression Omnibus (GEO, https://www.ncbi.nlm.nih.gov/geo/) was utilized to access scRNA-seq data pertinent to TNBC. This dataset, GSE161529, consists of 13 tumor-adjacent normal tissues: GSM4909253, GSM4909254, GSM4909257, GSM4909261, GSM4909263, GSM4909265, GSM4909266, GSM4909268, GSM4909270, GSM4909271, GSM4909272, GSM4909274, and GSM4909276, alongside 8 TNBC breast cancer tissue samples: GSM4909281, GSM4909282, GSM4909283, GSM4909284, GSM4909285, GSM4909286, GSM4909287, and GSM4909288. Subsequent data analysis was performed using the Seurat (v4.0.3) R package, which included normalization (LogNormalize), scaling (ScaleData), principal component analysis (PCA), nonlinear dimensionality reduction techniques (UMAP/t-SNE), and clustering and visualization of gene expression. Quality control measures were implemented based on the percentage of mitochondrial genes (<20%) and the number of expressed genes (200 < nFeature < 7000). Highly variable genes were selected based on their average expression and dispersion, as indicated by the variance-to-mean ratio.

### tSNE clustering analysis

To reduce the dimensionality of the scRNA-seq dataset, we performed PCA on highly variable genes, specifically focusing on the top 2000 genes exhibiting the greatest variance. The elbow method, implemented within the Seurat package, was utilized to select the top 30 principal components for subsequent analyses. Using a resolution of 0.8, we applied the “FindClusters” function provided by Seurat to identify major cell subpopulations. Following this, the t-SNE algorithm was employed for nonlinear dimensionality reduction of the scRNA-seq data. Additionally, the Seurat package facilitated the identification of marker genes for various cell subpopulations by annotating cells with lineage-specific marker genes.

### CytoTRACE analysis

Initially, the number of genes exhibiting detectable expression in each cell was quantified. Subsequently, the top 200 genes that demonstrated the highest correlation with Gene Counts were identified. The geometric mean of the expression levels of these 200 genes was calculated to establish the Gene Count Signature (GCS). This GCS was then smoothed based on the similarity among cells. The resulting values were ranked and normalized to a scale between 0 and 1, reflecting the predicted relative differentiation states of the cells; a value of 0 indicates a more differentiated state with reduced stemness, whereas a value of 1 indicates a less differentiated state with increased stemness.

### AUCell analysis

The gene set data and scRNA-seq data were analyzed using the “aucell_buildrankings” function to rank genes for each individual cell, resulting in a ranking matrix. In instances where genes exhibited identical expression values, a random ranking method was employed. Subsequently, the “aucell_calcauc” function was utilized to compute the Area Under the Curve (AUC) value for the gene set in each cell. The resulting AUC scores serve as indicators of the relative expression levels of the corresponding gene signature.

### Calculation of the stemness index and analysis of overall survival

The stemness features were identified using the OCLR machine learning algorithm with leave-one-out cross-validation, based on the mean RNA-Seq data of pluripotent stem cells obtained from the PCBC database (syn2701943). Subsequently, the normalized expression matrix of TNBC samples underwent Spearman correlation analysis with stemness markers, and the correlation coefficients were scaled between 0 and 1 to derive the mRNAsi. TNBC patients from the TCGA were categorized into high mRNAsi and low mRNAsi subgroups according to the median mRNAsi. Unsupervised consensus clustering was conducted based on the stemness classification, with subsampling of 80% of the entries repeated 1000 times. The k-means algorithm was employed to partition each subsample into several groups. The Kaplan-Meier (K-M) curve was utilized to assess the overall survival of the dry subtype.

### mIHC detection

Initially, the tissue sections were stained with hematoxylin and eosin (HE) to evaluate tissue quality, confirming the presence of TNBC tumor tissue alongside adjacent normal tissue. Subsequently, the blank tissue sections were treated according to the protocol of the Sextuple-Fluorescence immunohistochemical mouse/rabbit kit (Immunoway, USA, RS0039), which included sequential antigen retrieval (Immunoway, USA, YS0004), primary antibody incubation, and HRP-secondary antibody incubation. Following this, 100 µL of Tyramide working solution, labeled with fluorescent dyes 488, 647, and 580, was applied to the tissue, with DAPI added for mounting and imaging. The formula for average fluorescence intensity is as follows: average fluorescence intensity = total fluorescence intensity of the area/area. Additionally, Supplementary Table 2 provides details on the antibodies utilized in the mIHC experiment.

### Statistical analysis

Flow cytometry results were analyzed using Kaluza Analysis Software version 2.1. Tumor metastasis was assessed with Living Image® version 4.5.1 Software. Results from the mouse multi-factor panel were evaluated using Milliplex Analyst version 5.1. The R² values of all ELISA standard curves exceeded 0.99, and all experiments were conducted at least three times. Representative images of flow cytometry, immunohistochemical staining, immunofluorescence staining, and western blot analysis are presented. Data are expressed as mean ± SEM and analyzed using GraphPad Prism version 9. Differences between two groups were assessed using the unpaired, two-tailed Student’s t-test, while comparisons among multiple groups were performed using one-way ANOVA. The *p*-values were considered statistically significant at the following thresholds: *p < 0.05, **p < 0.01, ***p < 0.001, and ****p < 0.0001. Additionally, protein gene data for MDSC exosomes were obtained from the ProteomeXchange database, and gene data for metastatic breast cancer models were retrieved from NCBI GEO using the keywords “breast cancer” and “metastasis,” which were subsequently analyzed via the bioinformatics website (https://www.bioinformatics.com.cn/). All primers, antibodies, recombinant proteins, and inhibitors utilized in this study are detailed in the Supplementary Table.

### Ethics approval and consent to participate

All animal experimental protocols and care were conducted in accordance with the guidelines and standards established by the Experimental Animal Ethics Committee of Xinjiang Medical University. Triple-negative breast cancer tissue specimens were obtained from the Pathology Department of the Xinjiang Uygur Autonomous Region People’s Hospital, with approval from the Ethics Review Committee.

## Supplementary information

Supplementary Fig.s

Supplementary Materials

Uncropped Western Blot

## Data Availability

The data supporting the conclusions of this paper are provided within the paper itself, as well as in the TCGA and GEO databases. Furthermore, all data for this study are available from the corresponding author upon reasonable request.
