# Supplementary material for: PMN-MDSCs-derived exosomal S100A9 drives breast cancer progression by enhancing cancer stemness and CXCL5-mediated metastatic potential
Source: Cell Death Discov. 2026 May 18;12:293. doi: 10.1038/s41420-026-03134-7 (PMC13346805; doi:10.1038/s41420-026-03134-7)
Supplement: Supplementary file 1 — Supplementary Fig.s [file 41420_2026_3134_MOESM1_ESM.docx]

**Supplementary Figure**

**Figure S1**


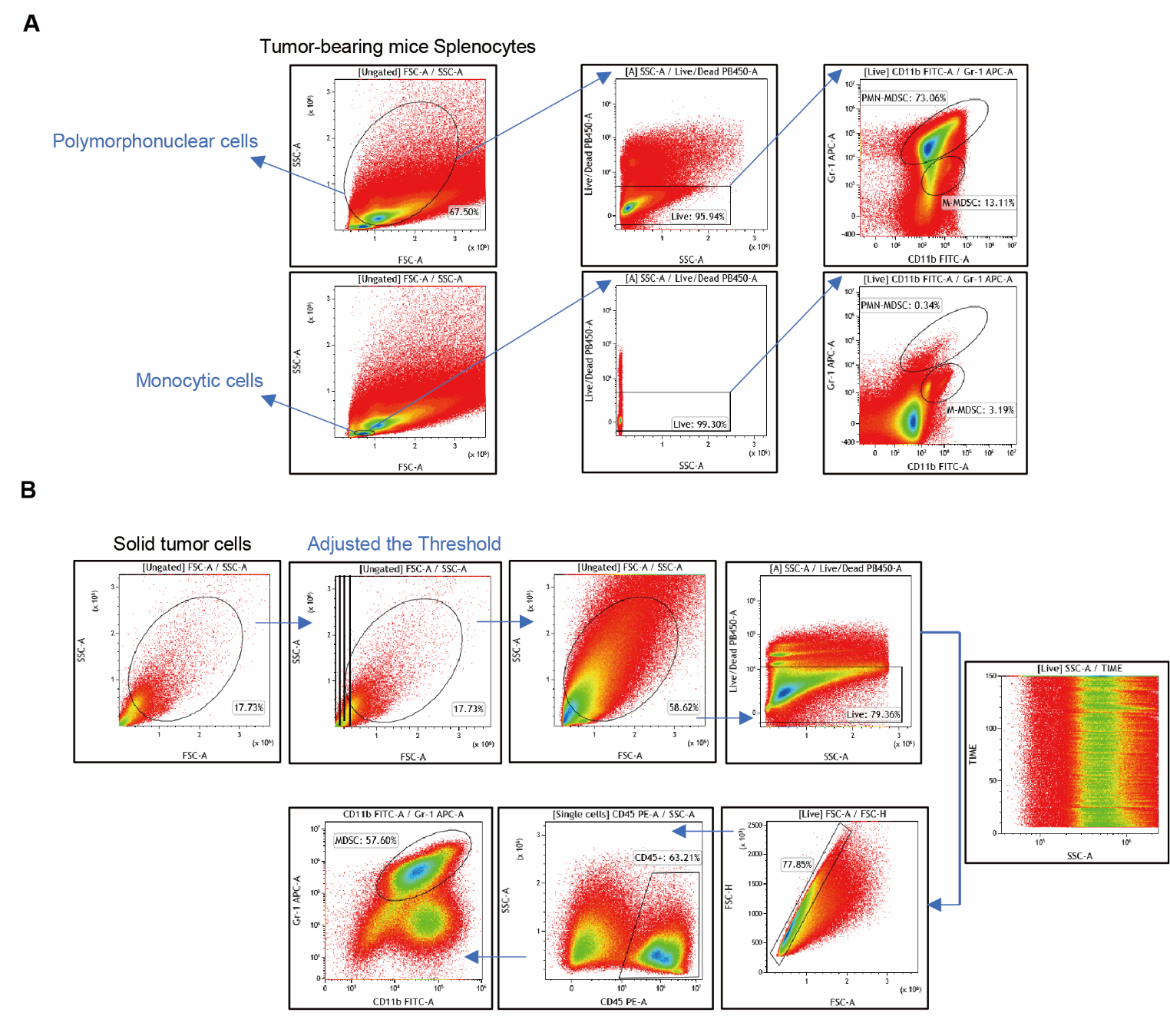


**Figure S1: The flow sorting scheme for PMN-MDSCs. (**A**)** After gating the polymorphonuclear and mononuclear cell populations within the splenocytes of breast cancer mice, we analyzed the PMN-MDSCs and M-MDSCs cell populations. (B**)** By adjusting the threshold of the sorting flow cytometer, we obtained flow cytometry plots for sorting MDSCs from the single-cell suspension derived from tumor tissue.

**Figure S2**


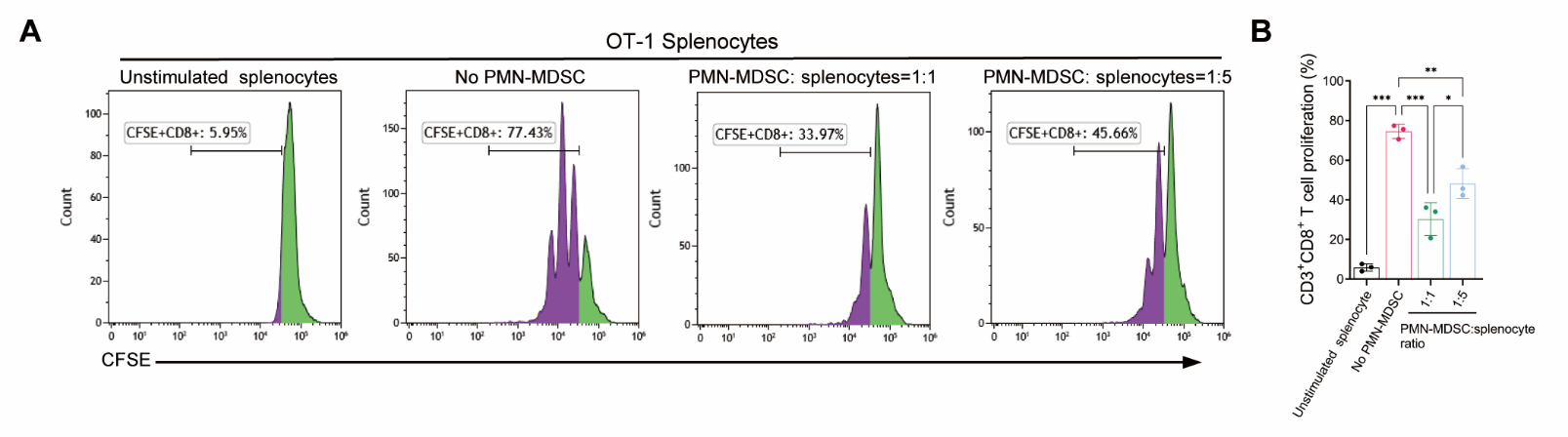


**Figure S2: The inhibitory effect of PMN-MDSCs on T cell function.** The flow histogram (A) and bar graph (B) depict the proliferation of CD8^+^ T cells within the splenocytes of OT-I mice that were stimulated with the SIINFEKL peptide. The results include experiments in which splenocytes were cultured both in isolation and in co-culture with varying ratios of PMN-MDSCs. One-way ANOVA followed by Tukey's post-hoc test was used for the analysis of multiple sample groups. **p* < 0.05, ***p* < 0.01, ****p* < 0.001.

**Figure S3**


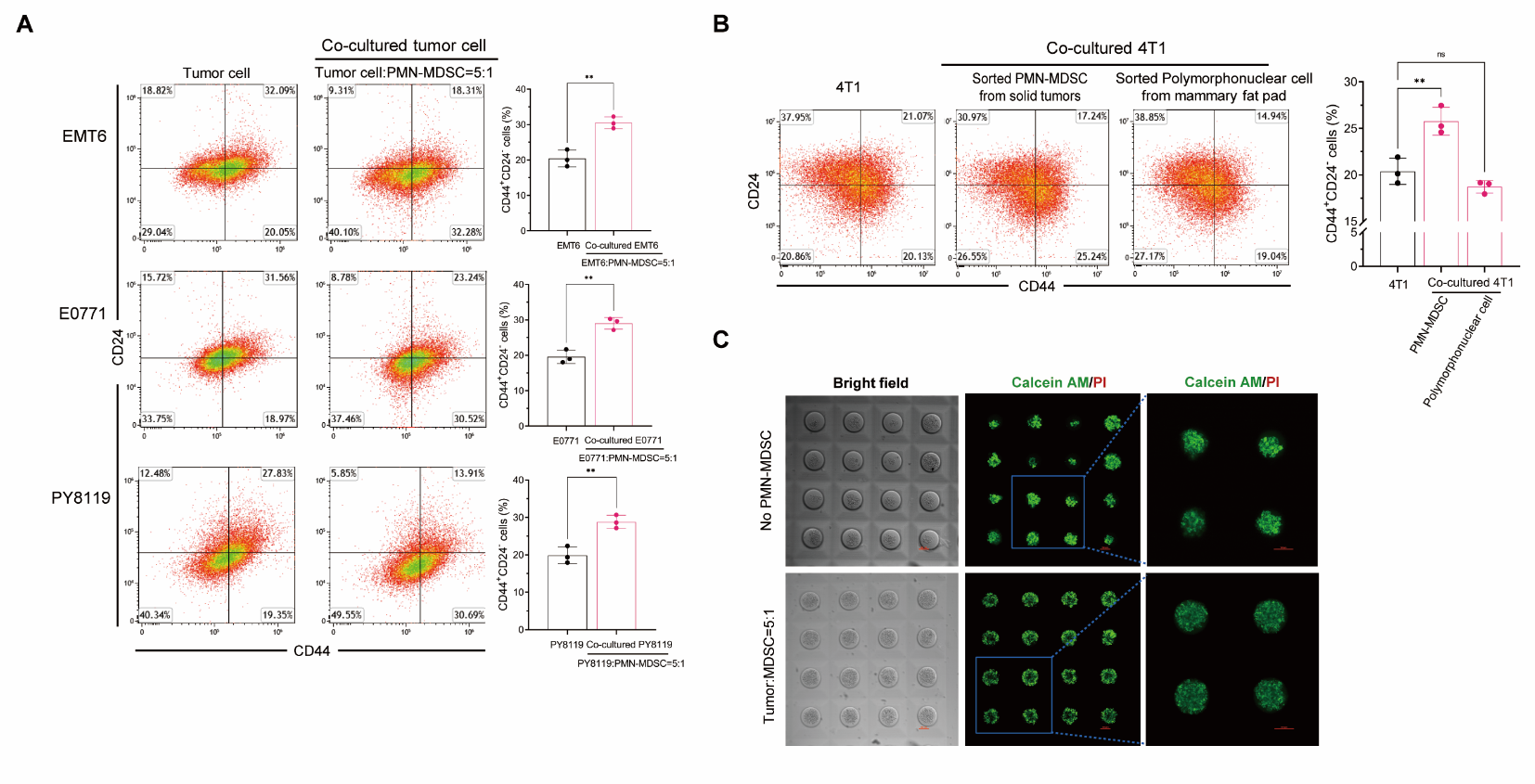


**Figure S3: PMN-MDSCs significantly enhance the proportion of CD44^hi^CD24^lo^ cell populations in 4T1 cells and facilitate tumor spheroid formation within the Chip matrix. (**A) Flow cytometry plots and bar graphs illustrate the CD44^hi^CD24^lo^ cell populations of 4T1 cells co-cultured with sorted polymorphonuclear cells obtained from the mammary fat pad tissue of healthy mice, as well as sorted PMN-MDSCs from mammary tumor-bearing mice, along with their statistical results. (B) Flow cytometry plots and corresponding bar graphs depict the CD44^hi^CD24^lo^ cell populations of EMT6, EO771, and PY8119 cells co-cultured with PMN-MDSCs. (C) Observations of tumor spheroid formation within the Chip matrix are presented, showcasing bright-field and Calcein-AM/PI staining images of 4T1 cells co-cultured with PMN-MDSCs. Scale bar: 100 µm. The Student's t-test is employed for the analysis of two sample groups, while one-way ANOVA followed by Tukey's post-hoc test was used for the analysis of multiple sample groups. ***p* < 0.01, with 'ns' indicating no significance.

**Figure S4**


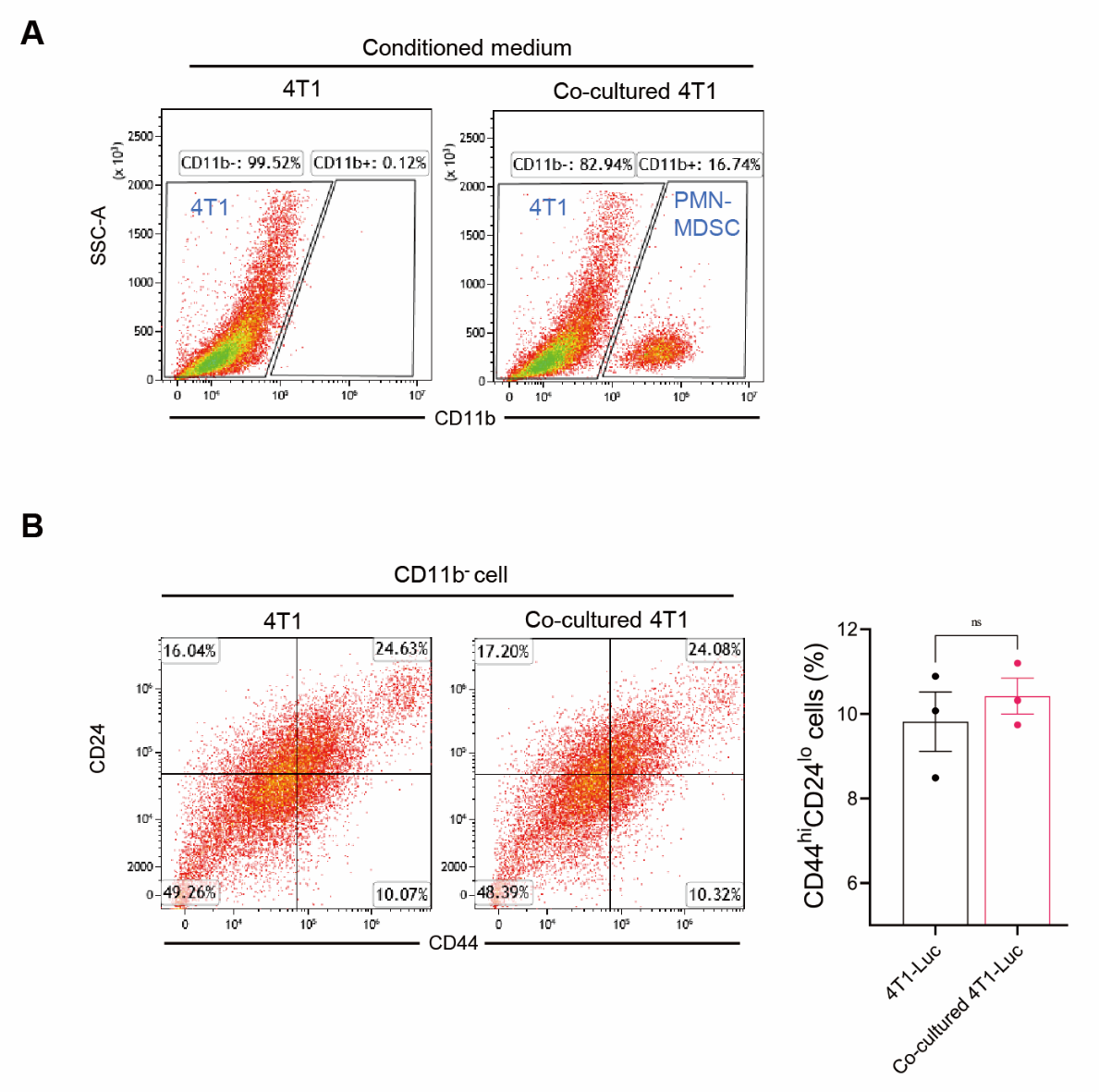


**Figure S4:** **The percentage of CD44^hi^CD24^lo^ subpopulation in 4T1 cells derived from the conditioned medium (CM). (**A) The flow cytometry plot illustrates the expression of CD11b in cells collected from the conditioned medium. CD11b^-^ cells represent 4T1 cells, while CD11b^+^ cells correspond to PMN-MDSCs. (B) The flow cytometry plot depicting the proportion of CD44^hi^CD24^lo^ cells within the CD11b^-^ population is accompanied by a statistical graph in the right panel. The Student's t-test is employed for the analysis of two sample groups, with 'ns' indicating no significance.

**Figure S5**


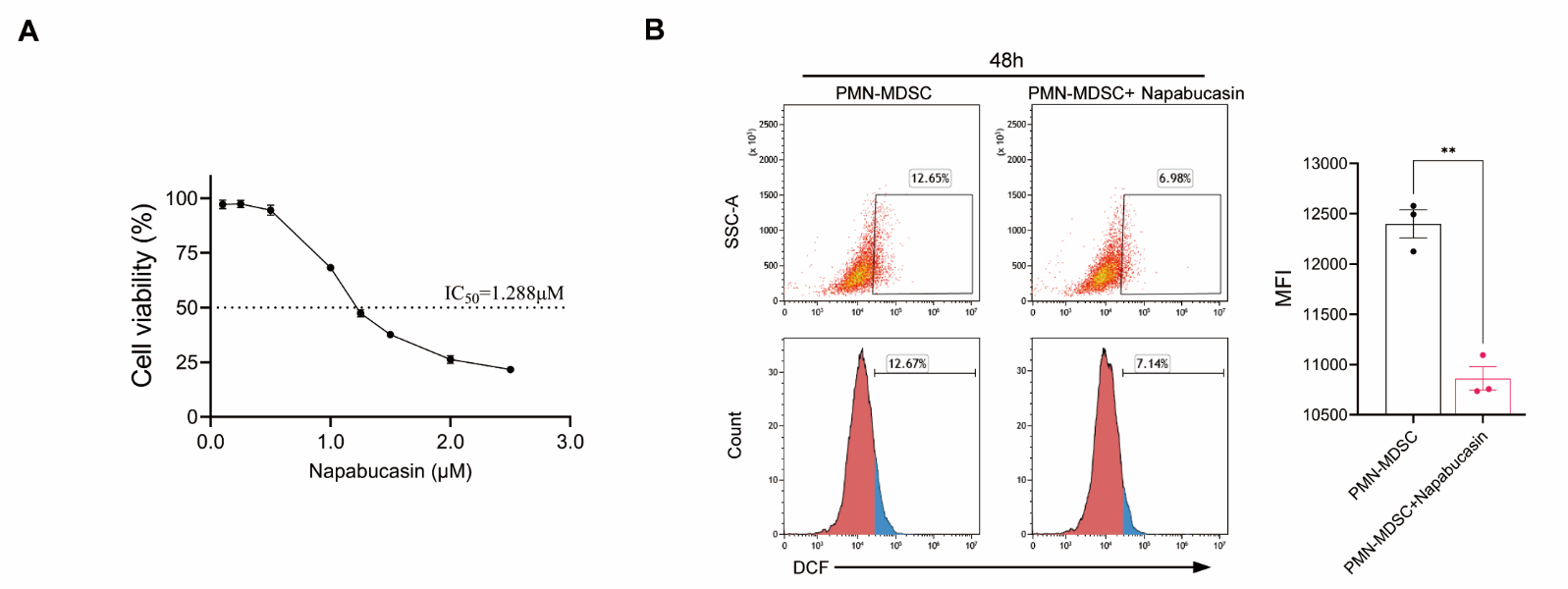


**Figure S5:** **The inhibitory effect of Napabucasin on the activity of 4T1 cells and PMN-MDSCs. (**A) The graph displays cell viability and the IC_50_ value for 4T1 cells exposed to various concentrations of Napabucasin. (B) Flow cytometry plots (left panel) and corresponding statistical graphs (right panel) show the mean fluorescence intensity (MFI) of reactive oxygen species (ROS) in PMN-MDSCs treated with or without Napabucasin. The Student's t-test is employed for the analysis of two sample groups. ***p* < 0.01, with 'ns' indicating no significance.

**Figure S6**


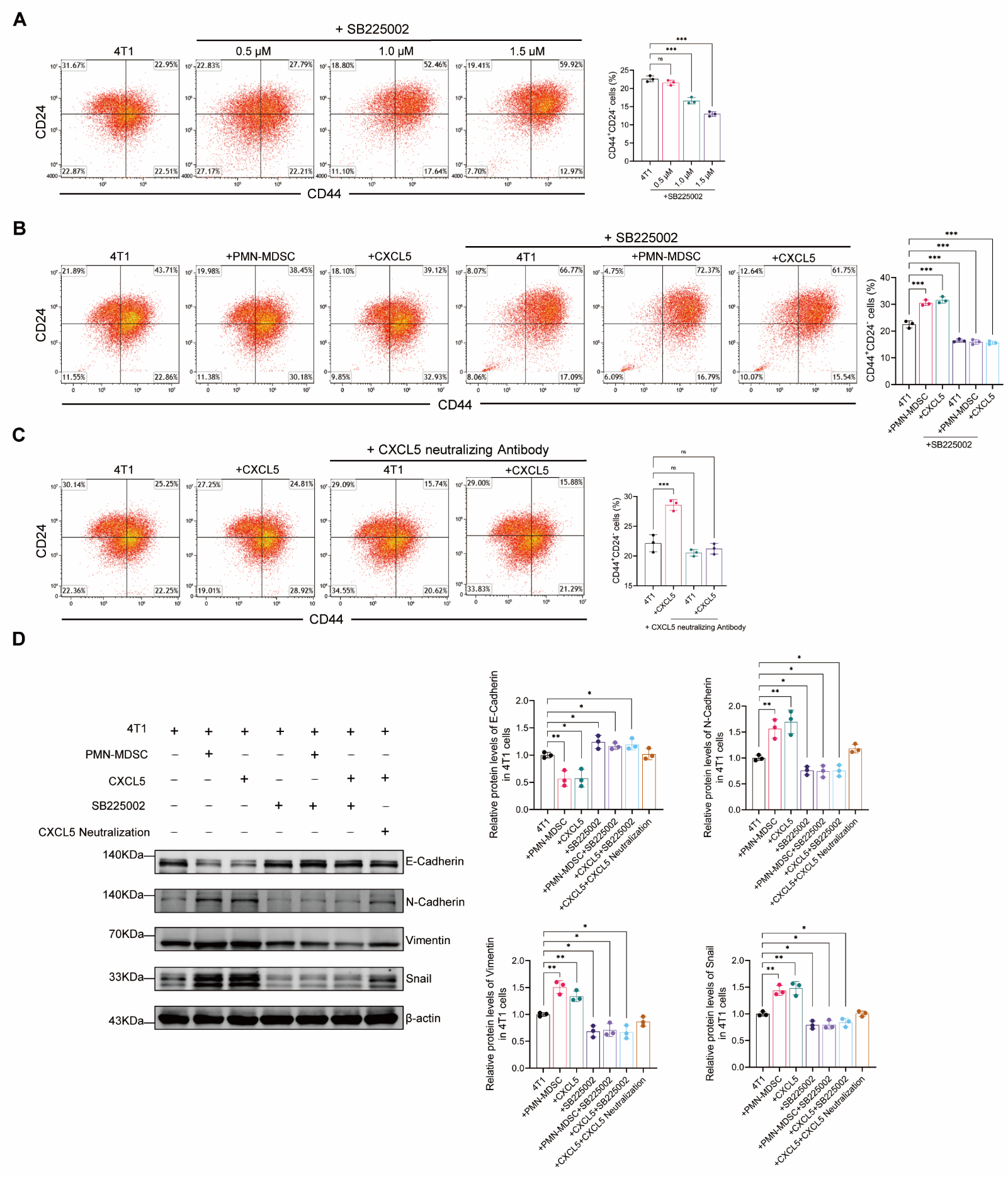


**Figure S6: The effects of the CXCR2 inhibitor SB225002 and the CXCL5 neutralizing antibody on the alterations in the CD44^hi^CD24^lo^ cell population in 4T1 cells. (**A) Flow cytometry results and corresponding statistical graphs depict the changes in the CD44^hi^CD24^lo^ cell population following the addition of varying concentrations (0.5 μM, 1.0 μM, 1.5 μM) of the CXCR2 inhibitor SB225002 to the 4T1 cell culture. (B) Flow cytometry and statistical graphs illustrate the alterations in the CD44^hi^CD24^lo^ cell population in 4T1 cells upon the introduction of 1.0 μM SB225002 to the culture system containing PMN-MDSCs or CXCL5. (C) Flow cytometry results after the addition of the CXCL5 neutralizing antibody to cultures of 4T1 cells, either alone or in combination with exogenous CXCL5. (D) The results of the protein immunoblotting experiments and their quantitative analysis demonstrate the expression levels of E-cadherin, N-cadherin, vimentin, and Snail in 4T1 cells influenced by PMN-MDSCs, CXCL5, SB225002, and CXCL5 neutralizing antibodies, either individually or in combination. One-way ANOVA followed by Tukey's post-hoc test was used for the analysis of multiple sample groups. The Student's t-test was employed for the analysis of two sample groups. **p* < 0.05, ***p* < 0.01, ****p* < 0.001, with 'ns' indicating no significance.

**Figure S7**


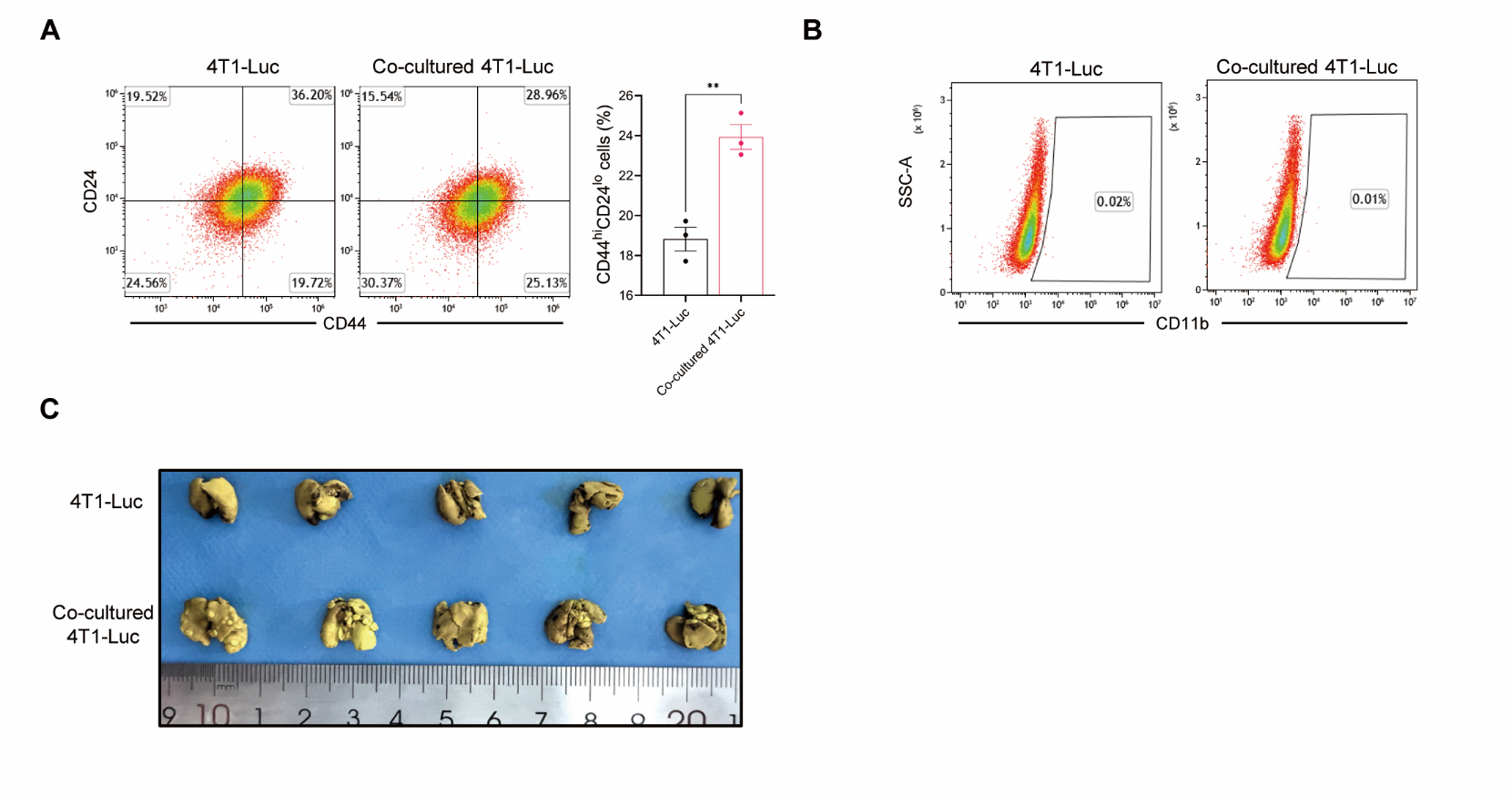


**Figure S7:** **Following co-culturing with PMN-MDSCs, 4T1-Luc cells demonstrate an enhanced capacity for tumor metastasis.** (A) The flow cytometry plots and statistical graphs depict the CD44^hi^CD24^lo^ cell populations in 4T1-Luc cells cultured either alone or co-cultured with PMN-MDSCs. (B) Flow cytometry plots illustrate the expression of CD11b^+^ cells (PMN-MDSCs) in 4T1-Luc cells that were intravenously injected into mice. (C) Lung tissues from breast cancer mouse models were treated with Bouin's fixative and photographed to illustrate their morphology and size. The Student's t-test is employed for the analysis of two sample groups. ***p* < 0.01.

**Figure S8**


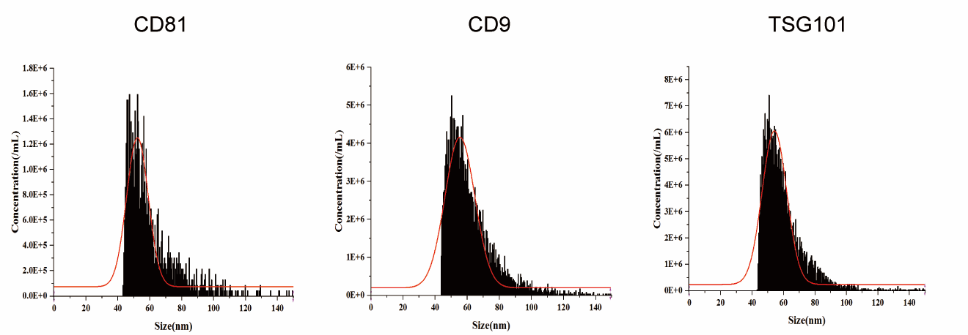


**Figure S8: The particle size distribution graph of exosomes derived from PMN-MDSCs (labeled with CD81, CD9, and TSG101) was detected using nanoflow cytometry.**

**Figure S9**


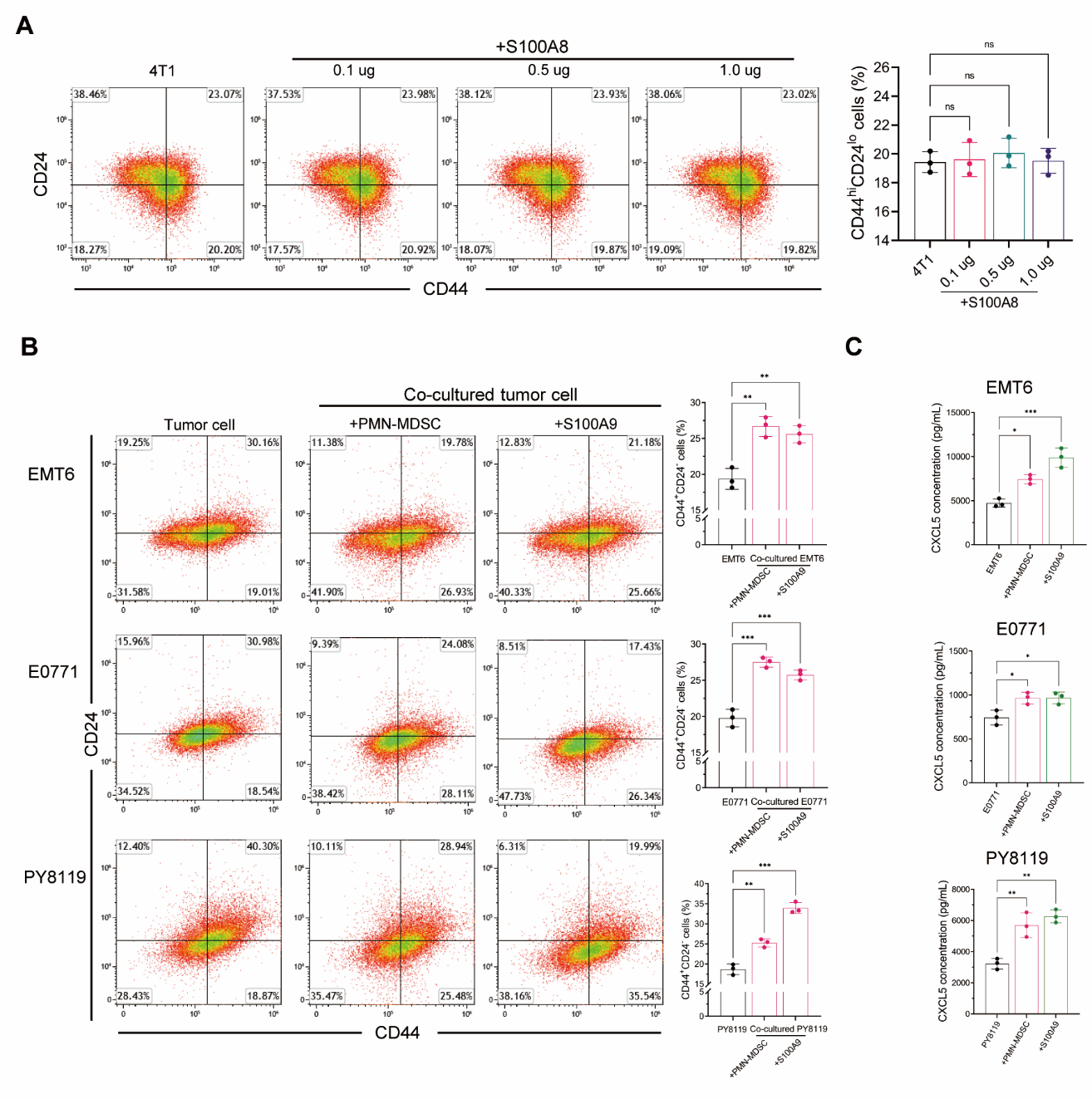


**Figure S9: The effects of S100A9 on stemness and CXCL5 secretion in various mouse breast cancer cell lines.** (A) Flow cytometry plots and statistical graphs illustrate the CD44^hi^CD24^lo^ cell population in 4T1 cells co-cultured with exogenous S100A8 at concentrations of 0.1 μg/ml, 0.5 μg/ml, and 1.0 μg/ml. (B) Flow cytometry plots (left panel) demonstrate the alterations in the CD44^hi^CD24^lo^ cell population of EMT6, EO771, and PY8119 cells following the addition of PMN-MDSCs or S100A9, while the statistical graphs (right panel) depict the proportion of the CD44^hi^CD24^lo^ cell population in EMT6, EO771, and PY8119 cells. (C) Statistical graphs illustrate the level of CXCL5 in the supernatant after the individual culture of EMT6, EO771, and PY8119 cells, as well as following the addition of PMN-MDSCs or S100A9. One-way ANOVA followed by Tukey's post-hoc test was used for the analysis of multiple sample groups. **p* < 0.05, ***p* < 0.01, ****p* < 0.001, with 'ns' indicating no significance.

**Figure S10**


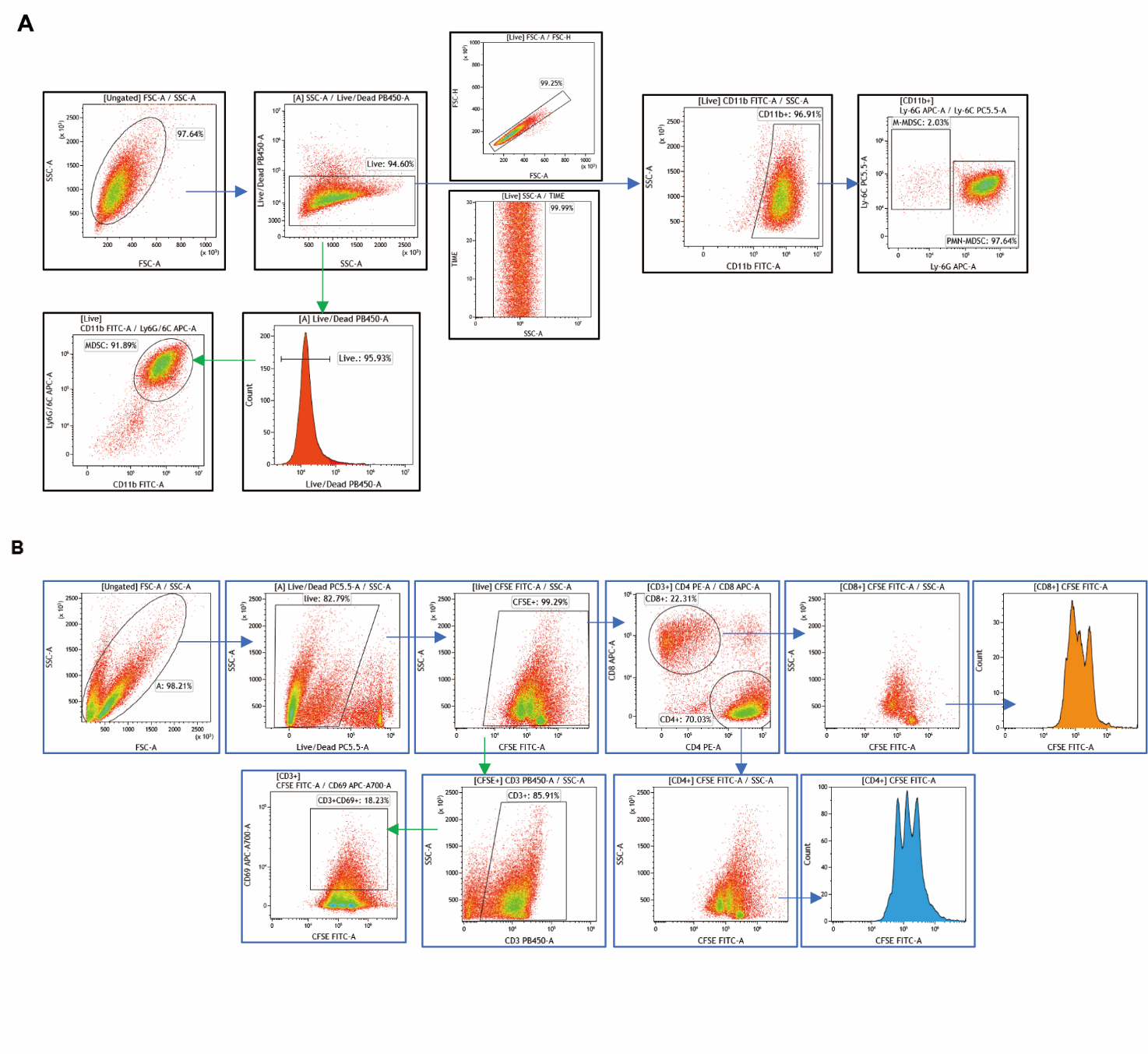


**Figure S10: Flow Cytometry Gating Strategies utilized in the present study. (**A) The flow cytometry gating strategy employed for identifying sorted MDSCs from a single-cell suspension prepared from tumors, which includes PMN-MDSCs and M-MDSCs, as well as for assessing their activity. (B) The flow cytometry gating strategy used for detecting the proliferation and activation of CD3^+^ T cells sorted using CD3/CD28 stimulation beads.
