# Supplementary material for: PMN-MDSCs-derived exosomal S100A9 drives breast cancer progression by enhancing cancer stemness and CXCL5-mediated metastatic potential
Source: Cell Death Discov. 2026 May 18;12:293. doi: 10.1038/s41420-026-03134-7 (PMC13346805; doi:10.1038/s41420-026-03134-7)
Supplement: Supplementary file 2 — Supplementary Materials [file 41420_2026_3134_MOESM2_ESM.docx]

**Table S1. Primer sequence information for qRT-PCR**

| Target | Forward (5'→3') | Reverse (3'→5') |
| --- | --- | --- |
| mCXCL11 | GGCTTCCTTATGTTCAAACAGGG | GCCGTTACTCGGGTAAATTACA |
| mCXCL10 | CCAAGTGCTGCCGTCATTTTC | GGCTCGCAGGGATGATTTCAA |
| mCXCL5 | TGCCCTACGGTGGAAGTCATA | TGCATTCCGCTTAGCTTTCTTT |
| mCCL20 | GCCTCTCGTACATACAGACGC | CCAGTTCTGCTTTGGATCAGC |
| mCXCR1 | TCTGGACTAATCCTGAGGGTG | GCCTGTTGGTTATTGGAACTCTC |
| mCXCR2 | ATGCCCTCTATTCTGCCAGAT | GTGCTCCGGTTGTATAAGATGAC |
| mOCT4 | CGGAAGAGAAAGCGAACTAGC | ATTGGCGATGTGAGTGATCTG |
| mSox2 | GCGGAGTGGAAACTTTTGTCC | CGGGAAGCGTGTACTTATCCTT |
| mNanog | TCTTCCTGGTCCCCACAGTTT | GCAAGAATAGTTCTCGGGATGAA |
| mALDH1A1 | ATACTTGTCGGATTTAGGAGGCT | GGGCCTATCTTCCAAATGAACA |
| mEpCam | GCGGCTCAGAGAGACTGTG | CCAAGCATTTAGACGCCAGTTT |

**Table S2. The antibody, recombinant protein and inhibitor information used in this study**

| **For flow cytometry staining** | | | | |
| --- | --- | --- | --- | --- |
| Antibody | Dilution | Catalog | | Manufacturer |
| Anti-mouse CD11b | 1:200 | 11-0112-82 | | invitrogen |
| Anti-mouse Ly6C | 1:50 | 45-5932-82 | | invitrogen |
| Anti-mouse Ly6G | 1:100 | 17-9668-82 | | invitrogen |
| Anti-mouse Ly6G/6C | 1:200 | 48-5931-82 | | invitrogen |
| Anti-mouse Ly6G/6C | 1:200 | 17-5931-82 | | invitrogen |
| Anti-mouse CD4 | 1:200 | 12-0041-82 | | invitrogen |
| Anti-mouse CD8 | 1:200 | 17-0081-82 | | invitrogen |
| Anti-mouse CD3 | 1:50 | 48-0031-82 | | invitrogen |
| Anti-mouse CD44 | 1:200 | 12-0441-82 | | invitrogen |
| Anti-mouse CD24 | 1:200 | 17-0242-82 | | invitrogen |
| Anti-mouse CD69 | 1:100 | 56-0691-82 | | invitrogen |
| Anti-mouse CD45 | 1:200 | 47-0451-82 | | invitrogen |
| Anti-mouse CD45 | 1:200 | 12-0451-82 | | invitrogen |
| DAPI | 1:400 | R37606 | | invitrogen |
| 7AAD | 1:400 | 00-6993-50 | | invitrogen |
| **For western blotting** | | | | |
| Antibody | Dilution | Catalog | | Manufacturer |
| Oct-4A | 1:1000 | 2840S | | Cell Signaling Technology |
| Nanog | 1:1000 | 8822S | | Cell Signaling Technology |
| Sox2 | 1:1000 | 14962S | | Cell Signaling Technology |
| ALDH1A1 | 1:1000 | 12035S | | Cell Signaling Technology |
| STAT3 | 1:2000 | 10253-2-AP | | Proteintech |
| Phospho-STAT3 | 1:1000 | 28945-1-AP | | Proteintech |
| CXCR2 | 1:1000 | 20634-1-AP | | Proteintech |
| CXCL5 | 1:1000 | DF9919 | | Affinity |
| E-cadherin | 1:20000 | 20874-1-AP | | Proteintech |
| Snail | 1:1000 | 3879S | | Cell Signaling Technology |
| BCL2 | 1:1000 | AG1222 | | Beyotime |
| Histone H3 | 1:2000 | 17168-1-AP | | Proteintech |
| N-cadherin | 1:2000 | 22018-1-AP | | Proteintech |
| Vimentin | 1:20000 | 10366-1-AP | | Proteintech |
| Phospho-Akt | 1:1000 | 9271T | | Cell Signaling Technology |
| Akt | 1:1000 | 9272S | | Cell Signaling Technology |
| Phospho-p44/42MAPK (Erk1/2) | 1:2000 | 4370T | | Cell Signaling Technology |
| p44/42 MAPK (Erk1/2) | 1:1000 | 9102S | | Cell Signaling Technology |
| CD81 | 1:1000 | 66866-1-Ig | | Proteintech |
| CD9 | 1:2000 | 20597-1-AP | | Proteintech |
| TSG101 | 1:2000 | 28283-1-AP | | Proteintech |
| Calnexin | 1:5000 | 10427-2-AP | | Proteintech |
| β-Actin | 1:5000 | HC201-01 | | TransGen Biotech |
| Goat Anti-Rabbit IgG (H+L), HRP Conjugate | 1:2000 | HS101-01 | | TransGen Biotech |
| Goat Anti-Mouse IgG (H+L), HRP Conjugate | 1:2000 | HS201-01 | | TransGen Biotech |
| HDAC1 | 1:1000 | AG8018 | | Beyotime |
| **For immunohistochemistry staining** | | | | |
| Antibody | Dilution | Catalog | | Manufacturer |
| Nanog Polyclonal antibody | 1:200 | 14295-1-AP | | Proteintech |
| SOX2 Polyclonal antibody | 1:200 | 11064-1-AP | | Proteintech |
| Ki67 Polyclonal antibody | 1:1000 | 28074-1-AP | | Proteintech |
| **For immunofluorescence staining** | | | | |
| Fluorescent dye | | | Catalog | Manufacturer |
| PKH 26 | | | HY-D1451 | MedChemExpress |
| Phalloidin-TRITC | | | Y249127-10μg | Beyotime |
| Hoechst 33258 | | | C1011 | Beyotime |
| **Recombinant Mouse protein** | | | | |
| Recombinant protein | | | Catalog | Manufacturer |
| Recombinant Mouse S100A8 protein | | | PDEM100028 | elabscience |
| Recombinant Mouse S100A9 protein | | | PDEM100021 | elabscience |
| Recombinant Mouse CXCL5 Protein, | | | TMPY-04790 | Targetmol |
| **For inhibition experiment** | | | | |
| Inhibitor | | | Catalog | Manufacturer |
| Napabucasin | | | 83280-65-3 | Targetmol |
| SB225002 | | | 182498-32-4 | Targetmol |
| CXCL5 neutralizing Antibody | | | MAB433-100 | R&D Systems |
| **For mIHC** | | | | |
| primary antibody | | | Catalog | Manufacturer |
| CD11B | | | HA722856 | HUABIO |
| CD15 | | | HA721246 | HUABIO |
| S100A9 | | | ET1702-73 | HUABIO |
| P-STAT3 | | | ET1607-39 | HUABIO |
| CXCL5 | | | HA722561 | HUABIO |
| CD44 | | | HA722845 | HUABIO |
