# Supplementary figures and images for: PMN-MDSCs-derived exosomal S100A9 drives breast cancer progression by enhancing cancer stemness and CXCL5-mediated metastatic potential
Source: Cell Death Discov. 2026 May 18;12:293. doi: 10.1038/s41420-026-03134-7 (PMC13346805; doi:10.1038/s41420-026-03134-7)

## Uncropped Western Blot

Fig.2C

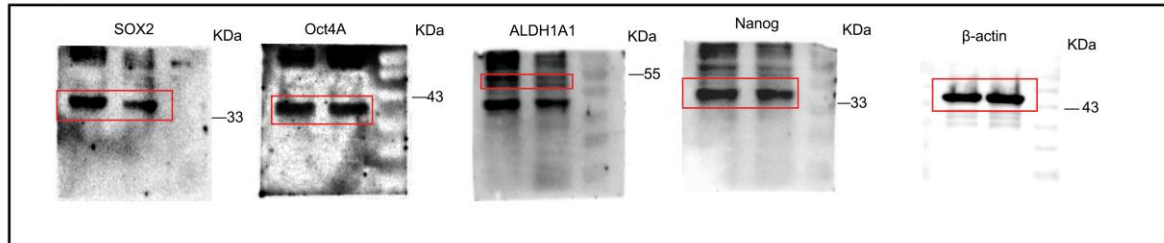

Fig.4A

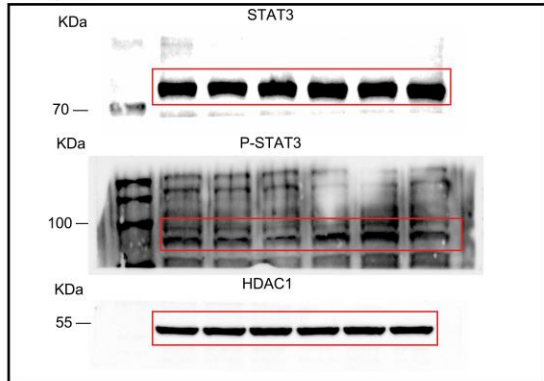

Fig.4B

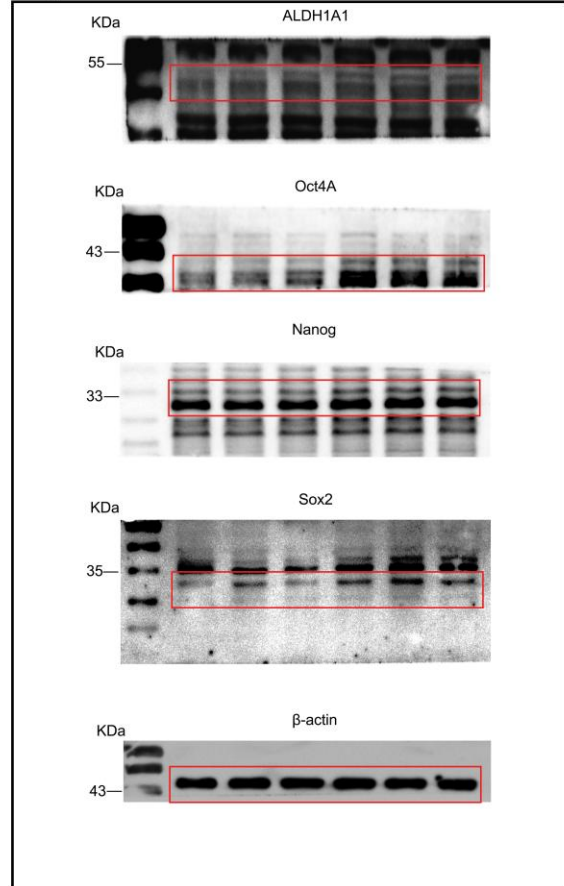

Fig.4I

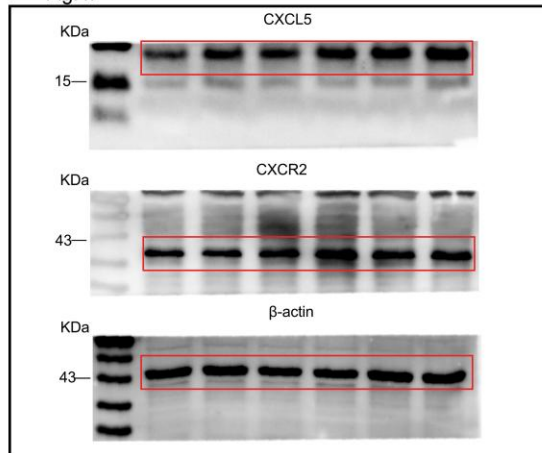

Fig.4C

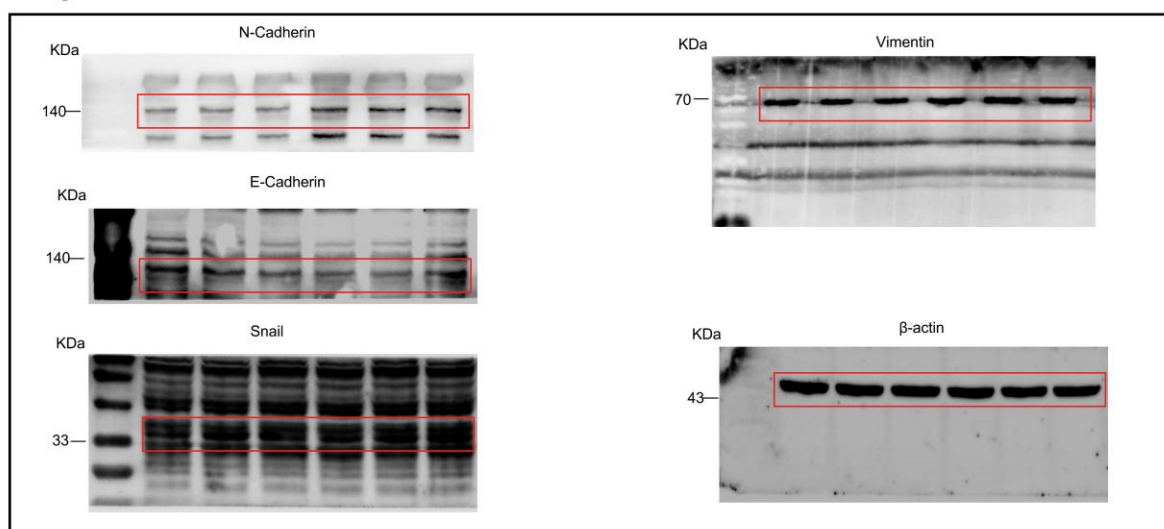

Fig.5C

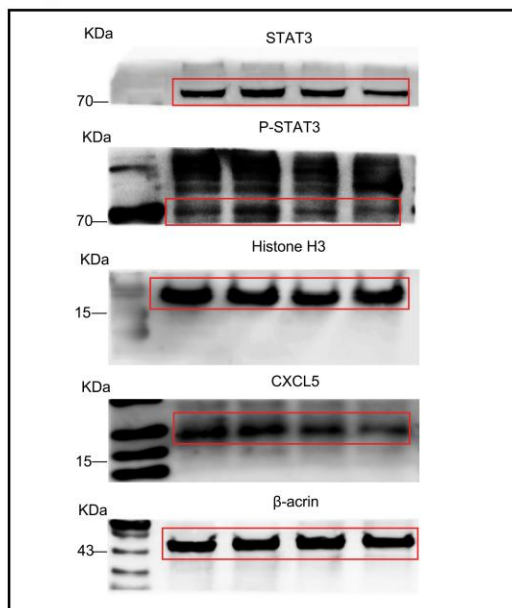

Fig.5E

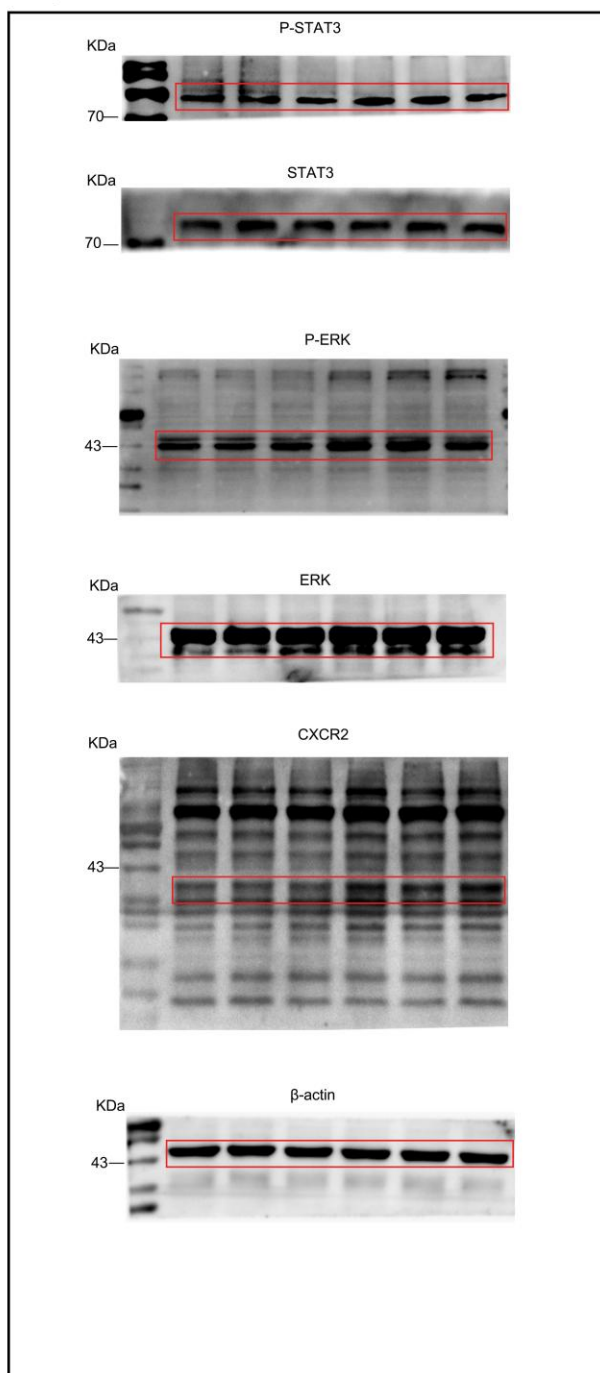

Fig.5F

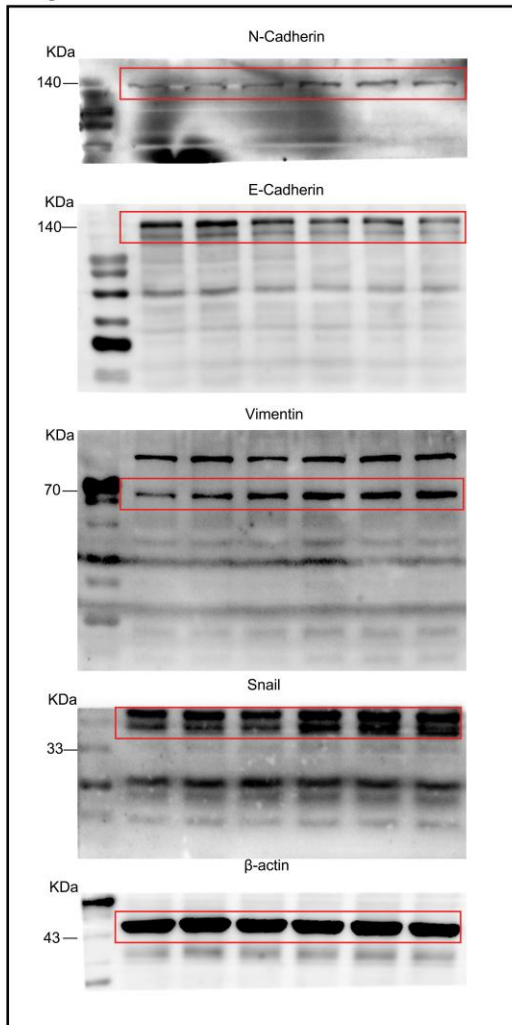

Fig.6E

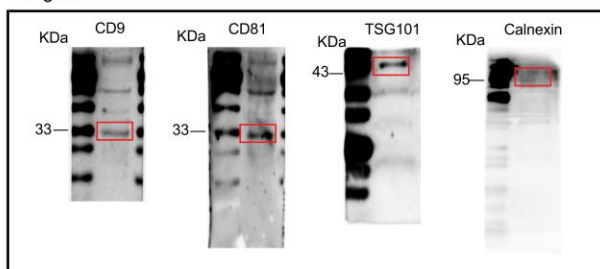

Fig.6H

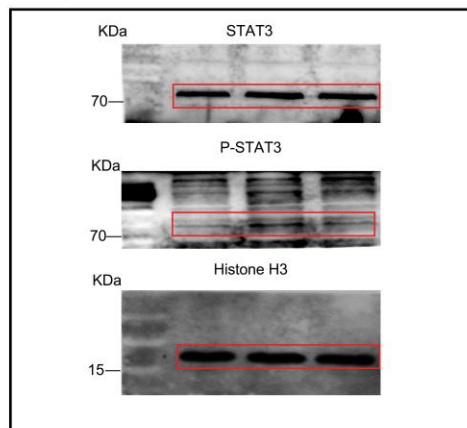

Fig.7E

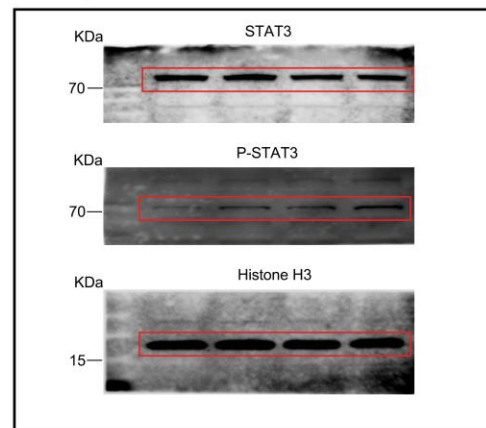

Fig.S6D

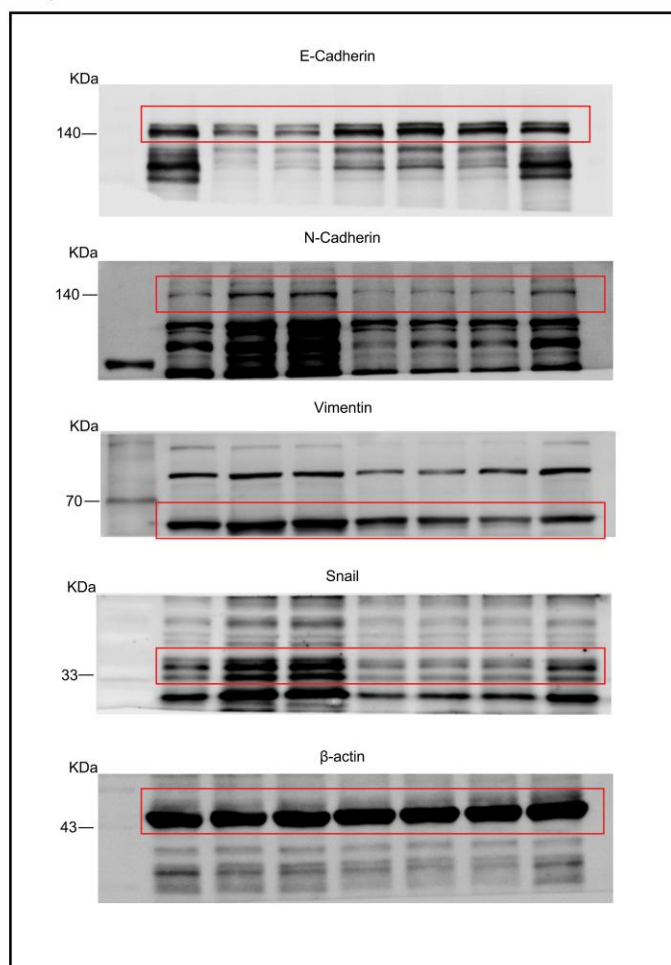

Supplement: Supplementary file 3 — Uncropped Western Blot [file 41420_2026_3134_MOESM3_ESM.pdf]
